# Computer Vision-Assisted Robotized Sampling of Volatile Organic Compounds

**DOI:** 10.1021/acs.analchem.4c03361

**Published:** 2024-09-26

**Authors:** Ching-Chi Chan, Noor Hidayat Abu Bakar, Chamarthi Maheswar Raju, Pawel L. Urban

**Affiliations:** Department of Chemistry, National Tsing Hua University, 101, Section 2, Kuang-Fu Rd., Hsinchu 300044, Taiwan

## Abstract

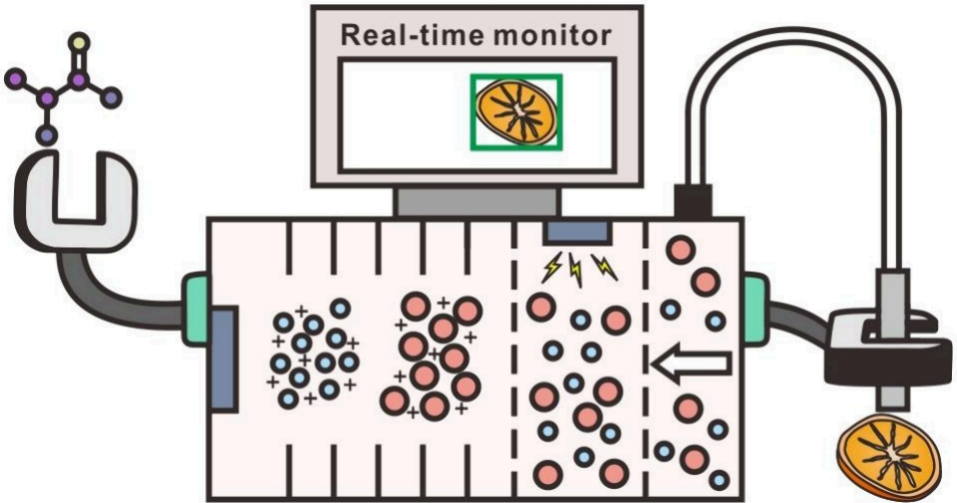

In conventional chemical analysis, samples are homogenized, extracted, purified, and injected into an analytical instrument manually or with a certain degree of automation. Such complex methods can provide superior performance in terms of sensitivity or selectivity. However, in some cases, it would be advantageous to possess a method that circumvents those preparatory steps, which require much attention. Here, we present a facile analytical approach to sampling volatile organic compounds (VOCs). Solid specimens emitting VOCs can be dropped onto the drop-off zone at a random position without any special alignment. A computer vision system recognizes specimen position, and a robotic arm places a sampling probe in the proximity of the specimen. The probe aspirates the VOCs—emitted by the specimen—with the aid of a suction force. A portion of the gaseous extract is transferred to the tritium-based ion source of a drift-tube ion mobility spectrometer. The ion mobility spectrum is immediately displayed in the customized graphical user interface (GUI). The sampling system also features a function for flushing extract ducts with hot nitrogen gas. Multiple specimens can be dropped for analysis at the same time. In one embodiment, the system can distinguish fresh meat from spoiled meat. When two meat specimens are placed on the drop-off zone, they are immediately sampled by the robotic arm, analyzed, and labeled on the digital image displayed on the GUI. Thus, the developed autosampling platform provides a hassle-free way of qualitative or semiquantitative analysis of raw specimens.

## Introduction

Ion mobility spectrometry (IMS) is a convenient technique for detecting gaseous analytes due to its speed, sensitivity, and low cost.^1,2^ IMS is commonly coupled with other techniques to analyze complex samples. For example, gas chromatography coupled with IMS is employed in clinical, food, and environmental analysis.^3^ Combining IMS with mass spectrometry (MS) enables separations of isomers, signal filtering, and untargeted annotations.^4^ Stand-alone ion mobility spectrometers have also been widely used for the past decades. They were occasionally coupled with a (micro)extraction step to extract analytes from real matrices.^5,6^ A number of research studies used atmospheric solid analysis probe (ASAP) as the sampling technique, which eliminates the need for sample preparation.^7−9^ The ASAP technique enables rapid direct analysis of solid and liquid samples.^10^

Sampling with hand-held probes is extensively employed in analytical chemistry.^11−13^ The recent advancements include the successful integration of hand-held probes with MS.^14−16^ This integration can be facilitated by solvent flow,^17,18^ gas flow,^16,19,20^ and laser beam^21^ to enable efficient transfer of analytes to the MS inlet. In some previous studies, the analyzed volatile organic compounds (VOCs) were also aspirated by a hand-held probe and detected by an IMS analyzer.^22,23^ By integrating such probes with a robotic arm, automated sampling can be achieved.^24^ For example, in a study by Abu Bakar et al., a robotized pen-probe was employed to conduct chemical mapping of VOCs, emanating from solid specimens by MS.^25^

Researchers have increasingly adopted robotized sampling to enhance the precision and reliability of their experimental setups.^26^ Implementation of robotized sampling can reduce the use of workspace,^27^ decrease sampling time,^28^ reduce human errors,^29^ increase throughput,^30^ enable flexible analysis,^31^ reduce cost,^32^ and enable continuous sampling.^33^ Furthermore, the high diffusivities and low concentrations of VOCs—emanating from the solid specimens—make it difficult to transfer all VOCs efficiently into the MS inlet.^34^ By employing a robotic system, VOCs can be systematically extracted from the sample matrices and subsequently transferred to the instrument automatically.^35^ This can increase the analytical throughput and reduce manual effort. While samples are usually fixed in a certain position within the robotic sampling system,^36−38^ computer vision (CV)-guided systems enable automated location of an object placed at a random position.^39^

CV is currently utilized in various fields.^40^ It provides several benefits, such as simplicity of use,^41^ high speed,^42^ nondestructive detection,^43^ and compatibility with portable systems.^44^ In recent years, CV has been utilized in analytical chemistry to process recorded images because color is the optical property of matter that may hold chemical information.^45^ CV—coupled with an analytical system—can monitor the progress of the reaction.^46,47^ A CV-guided robotic arm can pick and transfer the object to a desired location.^48^ Integrating CV with robotics enables the development of an automated CV-guided system.^49^

**Figure 1:**
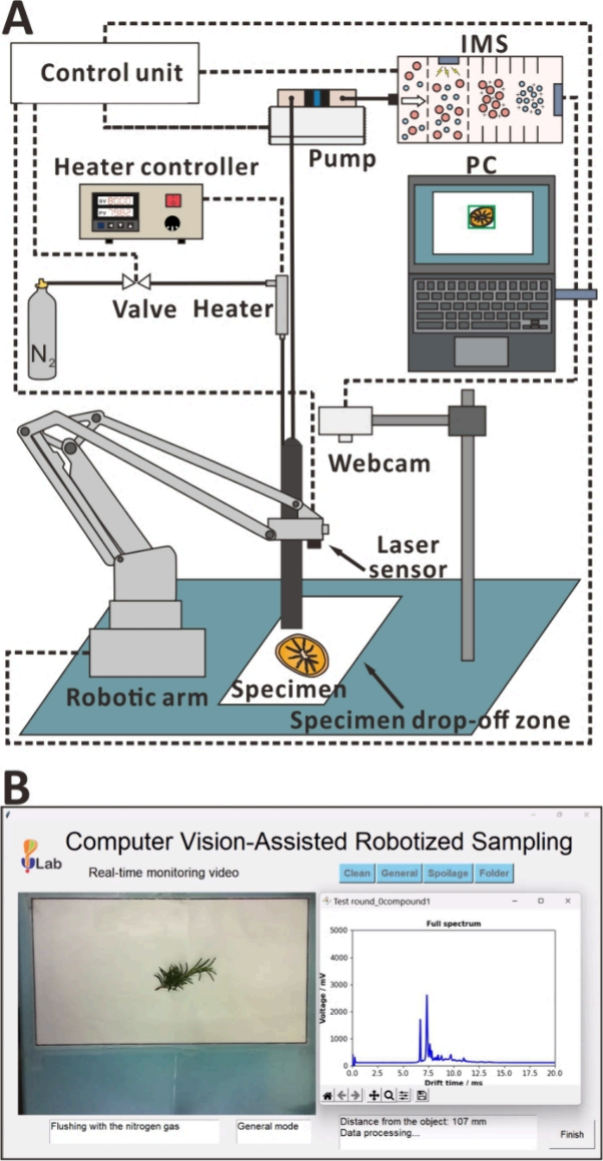
CV-guided robotic VOC sampling system: (A) layout and (B) screenshot of GUI.

In this study, we aimed to develop a system that takes advantage of CV and a robotic arm to sample VOCs from complex matrices (Figure 1). This methodology utilizes CV to precisely locate the target specimen, thereby facilitating the robotic arm to efficiently transfer VOCs to the inlet of the IMS instrument. The robotic system—assisted by CV—can autonomously locate target specimens and samples, even if their positions are random. The proposed sampling technique does not require solvent extraction or the use of sorbents. Furthermore, we demonstrated the capability of the developed system to analyze VOCs emanating from multiple objects. The resulting spectra are displayed in a graphical user interface (GUI).

## Experimental Section

### Chemicals and Samples

Water was purchased from Fisher Scientific (Pittsburgh, PA, USA). Methanol (anhydrous, 99.5+%) was purchased from Supelco (Kenilworth, NJ, USA). Ethanol (anhydrous, > 99.5%) was purchased from Echo Chemical (Miaoli, Taiwan). Ethyl acetate was purchased from Avantor (Philadelphia, PA, USA). (−)-Alpha-pinene, butyric acid, (R)(+)-limonene, and (S)(−)-nicotine were purchased from Alfa Aesar (Haverhill, MA, USA). Farnesene and trimethylamine were purchased from Sigma-Aldrich (St. Louis, MO, USA). (L)(−)-Carvone was purchased from Acros Organic (Geel, Belgium). Blue cheese, chicken breast, cotton T-shirt, incense, nicotine patches (containing 52.5 mg nicotine), salmon, shrimp, soy sauce, and tangerine were purchased in local stores (Hsinchu City, Taiwan). A rosemary plant and a mint plant were purchased in a local flower shop. Gasoline (unleaded 92) was purchased in a local gas station (Hsinchu City, Taiwan).

### VOC Sampling Setup

Here, we employed a robotic arm (uArm Swift Pro; UFactory, Guangdong, China) with a semicircular range of motion as a gaseous sample collection tool (Figure 1A). The positioning repeatability of the arm is 0.2 mm, and the maximum speed is 100 mm s^–1^. The pen-probe (diameter: 19 mm; length: 94 mm; material, aluminum; Figure S1) was designed using Inventor 2024 software (Autodesk, San Rafael, CA, USA) and fabricated by the NTHU workshop (Hsinchu, Taiwan). In the pen-probe, there are two channels connected with two tubings: one tubing (length: 200 mm; O.D.: 1/8 in.; I.D.: 1.02 mm; material: tetrafluoroethylene, TFE; cat. no. 58700-U; Sigma-Aldrich, St. Louis, MO, USA) for blowing the heated nitrogen gas to remove the carryover in the sampling system and another tubing (length: 600 mm; O.D.: 1/8 in.; I.D.: 1.02 mm; material: TFE; cat. no. 58700-U; Sigma-Aldrich, St. Louis, MO, USA) for drawing the analyte VOCs from the specimens by a diaphragm pump with a maximum flow rate of 34 L min^–1^ (cat. no. N840.3 FT.40.18; KNF, Trenton, NJ, USA).^50^ The diaphragm pump is resistant to chemicals, has a self-drying system to ensure that condensate is quickly removed from the pump, and has high pump efficiency when handling gas mixtures containing water vapor.^51^ The pen-probe was fixed to the robotic arm. The nitrogen gas (99.995%; Chiah Lung Enterprise Company, Hsinchu, Taiwan) was supplied from a cylinder at a pressure of ∼5 kgf cm^–2^ and was heated to 100 °C by a heater (electric heating tube; 150 V; 500 W; Ching Ta Heating Company, Taoyuan, Taiwan). A solenoid valve (normally closed; cat. no. UWS-08; Thai Xin Machinery, Kaohsiung, Taiwan) was placed between the gas cylinder and heater to control the heated nitrogen outflow during the washing time. Incorporating the tee junction (Union Tee 1/8; cat. no. SS-200–3; Swagelok, Solon, OH, USA) and poly(ether ether ketone) tubing with a 0.13 mm inner diameter (length: 80 mm; cat. no. JR-T-5999-M3; Valco Instruments, Houston, TX, USA) into the effluent flow line brought the flow rate to ∼48 mL min^–1^, which is below the IMS inlet limit of 50 mL min^–1^ (Figure S2).

### Imaging Setup

To capture the streaming video—shown in the GUI (Figure 1B)—a webcam (JW-03W; Jinpei, Taipei, Taiwan), with a resolution of 1920 × 1080 and a frame rate of 30 fps, was fixed above the central part of the specimen drop-off zone. A piece of matte laminated white paper (36 × 19 cm)—used to prevent light reflection—was adhered onto the acrylic board to delimit the specimen drop-off zone (Figure S3).

### Ion Mobility Spectrometry Setup

The IMS module (model: OEM-IMS; GAS, Dortmund, Germany) was equipped with a radioactive atmospheric pressure chemical ionization (R-APCI) source containing tritium, which emitted β particles with an initial activity of approximately 300 MBq. The module also included a 98 mm drift tube as the drift region and a Faraday plate as the detector. A moisture trap (catalog no. 20618; Supelco, Sigma-Aldrich) was used to remove water from the nitrogen gas supplied to the drift tube. The default instrumental parameters were as follows: drift voltage polarity, positive; injection pulse width, 150 μs; repetition rate, 20 ms; drift voltage, 240 V; blocking voltage, 120 V; injection voltage, 2500 V, aperture voltage, 0 V; drift gas flow rate, 150 mL min^–1^; and drift tube temperature, 80 °C. The drift tube in the ion mobility spectrometer was kept at 45 °C when idle. Putative identification of the VOCs recorded by IMS was conducted using a corona discharge APCI quadrupole time-of-flight (Q-ToF) mass spectrometer (LCMS-9030; Shimadzu, Kyoto, Japan).

### Object Detection

Object detection is a CV technique that typically employs machine learning to locate and identify specific objects within an image or video.^52^ It involves identifying the position and boundaries of objects while also categorizing them into different classes.^53^ The “you only look once” (YOLO) is the well-known one-stage machine learning model for object detection, capable of real-time analysis.^54^ The streamed video—shown in the analysis GUI during the experiment—is processed by the Python program using the OpenCV library along with the YOLOv5 algorithm. After starting the Python program, YOLOv5 is trained with a generic training file “yolov5s.pt”^55^ for 5 s, and the calibration GUI is called out. The robotic arm has to be calibrated by moving it to the initial position, which is the upper right corner vertex of the specimen drop-off zone. The initial position serves as both the starting and ending points throughout the measurements. Subsequently, the drop-off zone is defined by the four vertices of the rectangular white paper (placed on the acrylic board), which is mapped by the Python program. Afterward, the user presses the “Start” button to open the analysis GUI for displaying the experimental process in real time and initiate the autosampling process.

**Figure 2:**
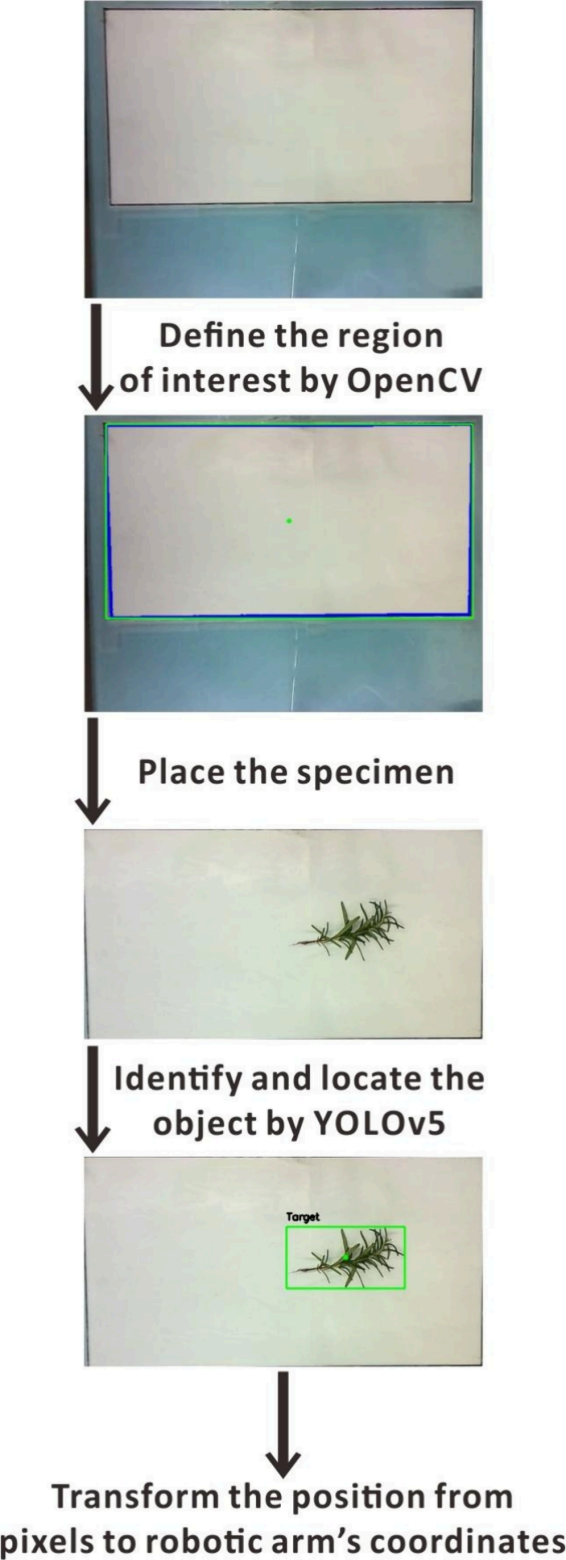
Workflow used for object detection.

In the field of object detection, candidate objects are initially selected. Subsequently, these candidates are screened to determine whether they are actual objects and categorized in one step. Multiple candidate boundary boxes initially cover a single object. However, adjusting the parameters of nonmaximum suppression^56^ helps eliminate redundant boxes and identify the optimal one. Each boundary box is described by the coordinates of the objects and the confidence score, which indicates the accuracy of classifying the object. Due to the random selection of real samples, a confidence score of 0.002 was set to ensure all objects are detected by the YOLOv5 algorithm. Various values were tested, and 0.002 was selected because it enabled the detection of the specimens analyzed in this study. In this case, there were several boundary boxes on the same object. To ensure that only one boundary box is on each object, we further adjusted the following parameters with different purposes: (1) Set the “Intersection over Union” (IOU) to 0 to prevent overlap between boundary boxes, where IOU represents the “intersection of two boundary boxes” divided by the “union of two boundary boxes”; (2) set class-agnostic to “true”, meaning prioritize the most accurate boundary box based solely on its location and confidence score, without considering the object class; and (3) set the “maximum number of detections per image” to 10. This approach reduces multiple detections of the same object and decreases the number of boundary boxes. However, due to the white color of the specimen drop-off zone, the zone itself is not recognized as an object. The YOLOv5 algorithm delimits the specimen with a boundary box. The left upper corner and right bottom corner coordinates of that box are averaged to obtain the central position of the object (Figure 2). In the streaming video, OpenCV draws a rectangle around the specimen and a circle at its center. Next, the real-time monitoring video is shown in the analysis GUI. After sampling, the picture and the corresponding spectrum are saved in a .png file.

Additional experimental details are included in the Supporting Information.

## Results and Discussion

### Optimization of the Autosampling Platform

Four standard compounds (ethyl acetate, limonene, pinene, and trimethylamine) were used to optimize the CV-guided autosampling platform for IMS. Ethyl acetate and trimethylamine were dissolved in water to concentrations of 4.0 × 10^–3^ and 4.4 × 10^–2^ M, respectively, while limonene and pinene were diluted in pure methanol to 6.0 × 10^–1^ and 4.0 × 10^–1^ M, respectively. Subsequently, 10 μL of sample solution was pipetted onto filter paper disks (Ø = 15 mm). The robotic arm moved toward the filter paper disk (mounted on a black tray), which was placed in the specimen drop-off zone. After the “Sampling” message appeared in the GUI, the pen-probe approached the filter paper for sampling.

In the IMS instrument used here, β particles—produced in the ion source—react with nitrogen gas in the instrument and form nitrogen ions.^57^ The nitrogen ions further react with moisture, forming protonated water clusters (H^+^(H_2_O)_*n*_), which are represented by the reactant ion peak in the ion mobility spectrum.^58^ When an analyte vapor is introduced, it reacts with protonated water clusters by proton transfer, hydride abstraction, charge transfer, and nucleophilic attachment,^59^ resulting in the formation of monomer, dimer, or trimer ions.^60^ The formation of protonated analyte clusters, which give rise to analyte ion peaks in the ion mobility spectrum, is dependent on the amount of analyte introduced into the ion source. Normally, the spectrum shows protonated monomers at low concentrations and protonated dimers at high concentrations.^23^

**Figure 3:**
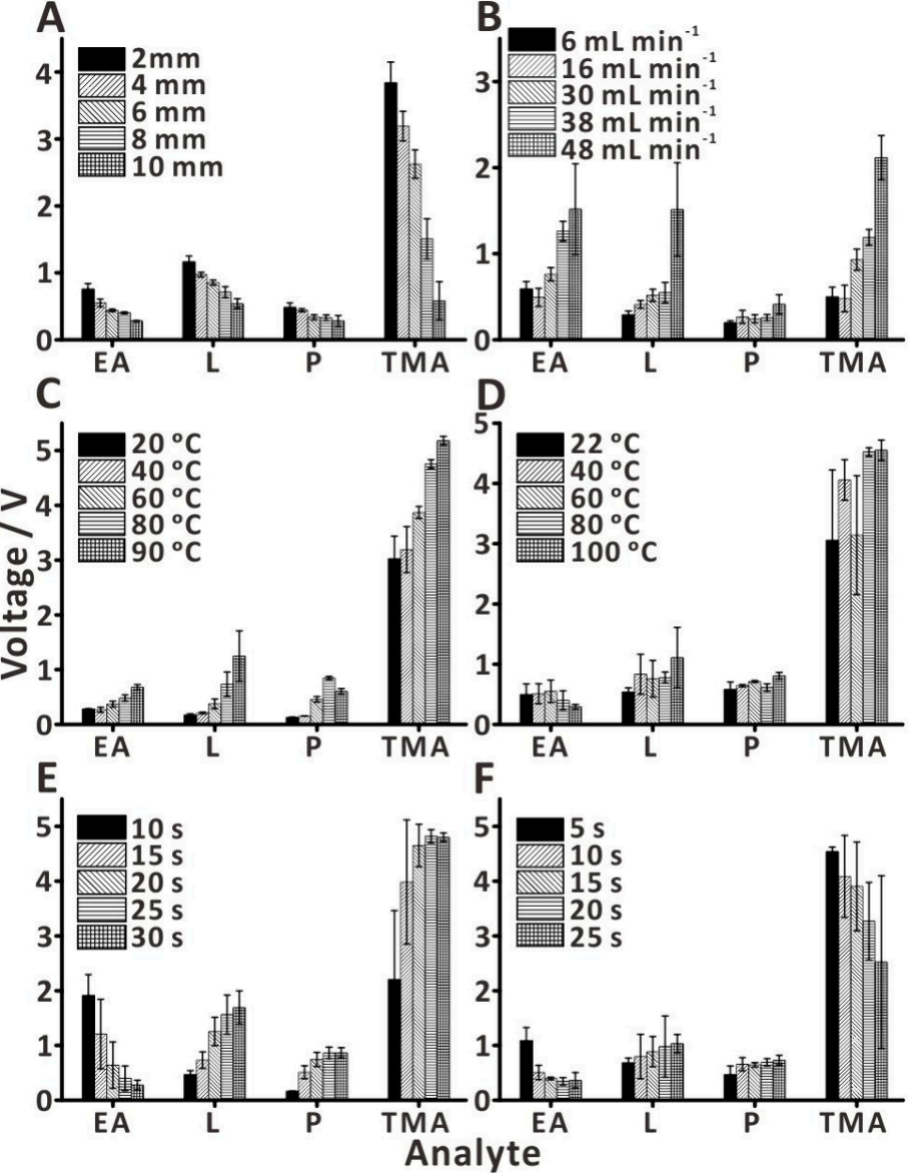
Optimization of the autosampling platform: (A) distance between the bottom of the pen-probe and the sample; (B) flow rate of the autosampling platform; (C) drift tube temperature; (D) heated nitrogen gas temperature; (E) sampling time; and (F) blank time. Labels: EA—ethyl acetate; L—limonene; P—pinene; and TMA—trimethylamine. Error bars represent standard deviation (*n* = 3).

To improve the performance of the autosampling platform, several parameters were optimized: distance between the bottom of the pen-probe and the sample, flow rate of the autosampling platform, drift tube temperature, heated nitrogen gas temperature, sampling time, and blank analysis (Figure 3). Peak intensity (maximum peak height) of the standard was determined as a function of the varied conditions. Variation of the distance between the pen-probe and the sample (Figure 3A)—ranging from 2 to 10 mm—significantly influences the signal intensity. Decreasing the sampling distance results in enhanced introduction of the gaseous sample into the IMS ion source, consequently increasing the signal intensity. This phenomenon can be explained by the gas diffusion coefficient in the air. For example, ethyl acetate exhibits a diffusion coefficient of 0.137 cm^2^ s^–1^ when dispersed in nitrogen gas at 355 K.^61^ Diffusion coefficients of gases are typically ∼100,000× higher than those of liquids, and lighter molecules have higher average speeds than heavy molecules at the same temperature.^62^ Therefore, the larger amounts of VOCs could be pumped into the IMS instrument over a shorter sampling distance, and 2 mm was chosen as the sampling distance to reduce the gas diffusion effect. Varying the flow rate of the autosampling platform—from 6 to 48 mL min^–1^—also affected the signals to a great extent (Figure 3B). The IMS inlet limit is 50 mL min^–1^, which is a very small flow rate for open-space sampling. If one were using the low flow rate pump without flow splitting, then there would not be a sufficient aspirating force to sample the VOCs from the sample. Therefore, to maximize VOC sampling efficiency, we chose the high flow rate diaphragm pump (34 L min^–1^), ensuring high aspirating force, and fitted it with a tee junction (Figure S2) to lower the autosampling platform flow rate. The drift tube temperature is also an important parameter in the IMS analysis, which affects the reactant ions in the spectrum.^63^ Increasing the drift tube temperature from 20 to 90 °C resulted in a corresponding increase in the signal intensity (Figure 3C). Although 90 °C provided the best performance, we selected 80 °C as the working temperature to avoid damaging the instrument, which has a temperature limit of only 100 °C.^64^ When the nitrogen gas temperature was varied from room temperature to 100 °C (Figure 3D), the signal intensities of the standards were slightly increased. This phenomenon is similar to the use of drying gas in MS, which facilitates the evaporation of the solution during analysis. While varying the sampling time from 10 to 30 s, the signal intensity was averaged over the final 5 s of the sampling period (Figure 3E). Although the sampling time and flow rate were the same, the signal intensities of limonene, pinene, and trimethylamine increased during the analysis, while the signal intensity of ethyl acetate rapidly decreased. The sampling time of 20 s was chosen due to the acceptable signal intensity achieved with the four standards. When varying the sampling time from 5 to 25 s (Figure 3F), the signal intensity of limonene and pinene slightly increased due to the greater evaporation of analytes. Ethyl acetate and trimethylamine, due to their low molecular weight and high volatility, resulted in lower amounts being sampled as the blank time increased. The blank time of 10 s was chosen due to the acceptable signal intensity achieved with the four standards. It should be noted that most performance optimizations converge for multiple analytes except for sampling time (Figure 3E). We believe this discrepancy is caused by differences in vapor pressures and proton affinities. For the final settings, see Table S1.

### Characterization of the Autosampling Platform

**Figure 4:**
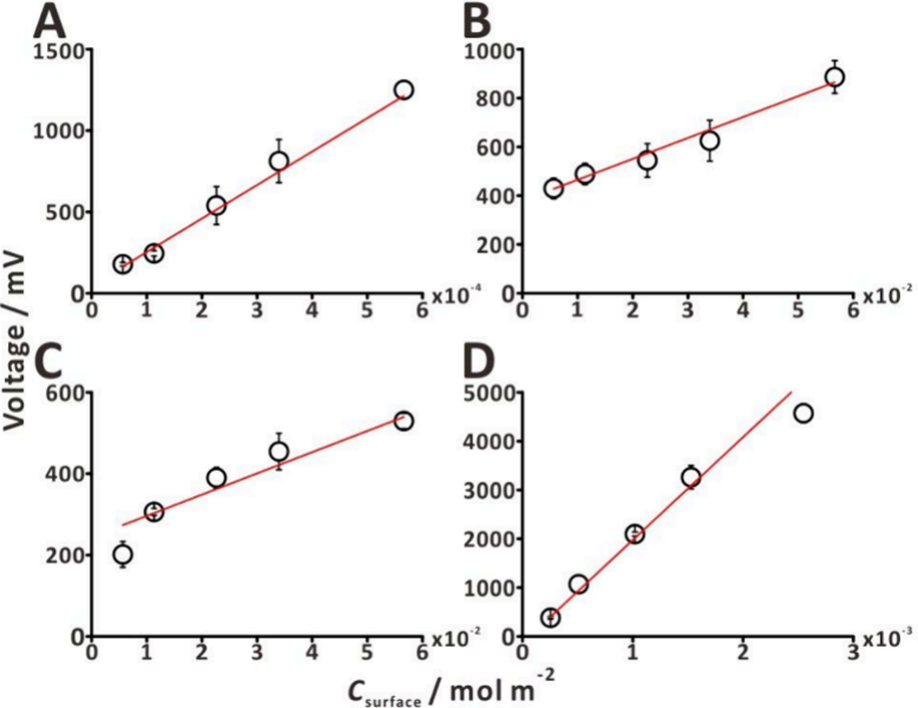
Calibration plots for the autosampling platform analysis of the selected test compounds: (A) ethyl acetate (drift time, 10.06 ms); (B) limonene (drift time, 8.97 ms); (C) pinene (drift time, 10.46 ms); and (D) trimethylamine (drift time, 7.07 ms). Five-level calibration ranges: from 10^–3^ to 10^–2^ M (ethyl acetate), from 10^–1^ M to 1 M (limonene), from 10^–1^ M to 1 M (pinene), and from 4.4 × 10^–3^ M to 4.4 × 10^–2^ M (trimethylamine). A 10 μL aliquot was deposited on a filter paper disk (Ø = 15 mm) and detected immediately. The analyses were conducted within 30 s (10 s blank and 20 s sampling). The average intensity was measured from 15 to 20 s (taking into account the ∼5 s travel time of VOCs from the probe to the ion source).

To characterize the CV-guided robotized autosampling platform, we have evaluated its repeatability and reproducibility and prepared calibration curves (Table S2 and Figure 4). We used four standard compounds to get the peak amplitude: ethyl acetate (4.0 × 10^–3^ M), limonene (6.0 × 10^–2^ M), pinene (4.0 × 10^–2^ M), and trimethylamine (4.4 × 10^–2^ M). These four compounds represent different classes of chemicals—esters, terpenes, and an amine—with varying volatilities, and they naturally occur in various sources. The repeatability (10 replicates on 1 day) and reproducibility (3 replicates on each of 6 out of 8 days) values for the four compounds were 5.9–17.0% and 6.5–14.0%, respectively. Note that the autosampling platform could not be operated reliably on some (e.g., rainy) days due to high ambient humidity (2 out of 8 days), which affected ionization giving rise to high moisture-related peaks (drift times, 6.83, 7.76, and 8.14 ms). In fact, the influence of moisture on APCI-IMS results was reported previously (cf. refs (63) and (65)). The calibration equations were based on the total amounts of standards deposited on the filter paper disk (mol m^–2^) versus the peak amplitudes (mV) of the analytes (Table S2 and Figure 4). The calibration data sets were fitted with linear functions. The *R*^2^ values for the four standards were in the range of 0.9497–0.9900. The limit of detection and limit of quantification were in the range of 3.28 × 10^–5^ to 1.10 × 10^–2^ mol m^–2^ and 9.94 × 10^–5^ to 3.32 × 10^–2^ mol m^–2^, respectively.

### Application of the Autosampling Platform in the Analysis of Real Specimens

**Figure 5:**
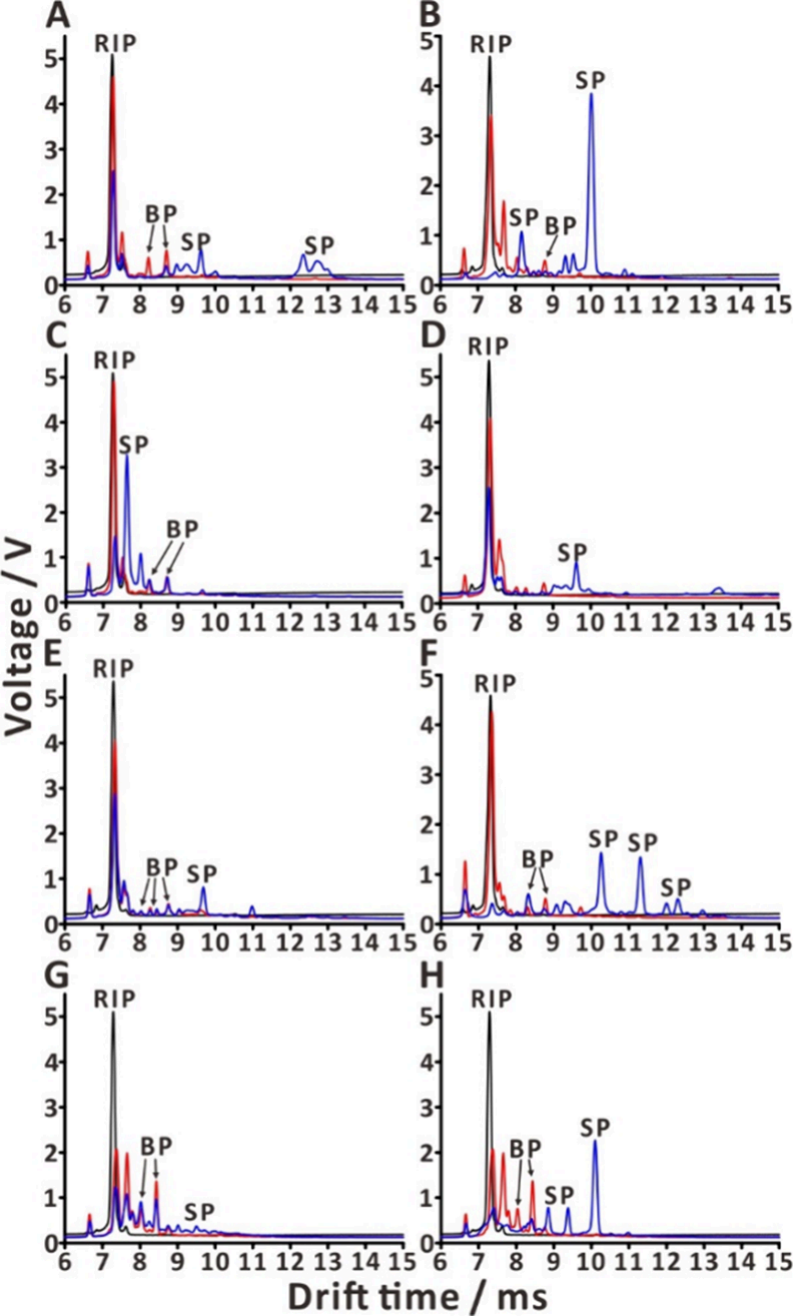
Representative results obtained using the autosampling platform: (A) tangerine peel; (B) nicotine patch; (C) soy sauce; (D) rosemary sprig; (E) mint sprig; (F) blue cheese; (G) fabric exposed to incense smoke; and (H) gasoline. Lines: black—instrumental blank; red—system blank; blue—real specimen. Labels: RIP—reactant ion peak; BP—blank peak (related to the system, e.g. contaminant); SP—sample peak.

The developed system was further tested with several real specimens in two applications. The first application was demonstrated using the “General mode”. A real specimen (tangerine peel, nicotine patch, soy sauce on the filter paper disk, rosemary sprig, mint sprig, blue cheese, fabric exposed to incense smoke, and gasoline on the filter paper disk) was placed in the specimen drop-off zone and immediately analyzed (Figures 5 and S4, Movie S1). The corresponding ion mobility spectrum was displayed in the GUI. For example, the ion mobility spectrum of tangerine peel shows a dimer of limonene (drift time, 12.34 ms) (Figure 5A), with additional peaks at 8.97 ms (monomer) and 12.71 ms (trimer; cf. ref (66)) also observed in a limonene standard (Figure S5A). In fact, limonene has been reported to account for ∼45–90% of the total terpenoids in tangerines.^67^ The analysis of the nicotine patch led to recording of peaks at 8.75 and 10.02 ms (Figure 5B), which correspond to the ethyl acetate monomer and dimer, respectively (Figure S5B). In fact, ethyl acetate is one of the solvents used in the adhesive material of the nicotine patch.^68,69^ Analysis of soy sauce led to recording the monomer peak of ethanol at 7.64 ms (Figure 5C). This peak overlaps with the peak of pure ethanol (drift time, 7.64 ms; Figure S5C). The presence of ethanol in soy sauce was expected.^70,71^ It is a byproduct of fermentation of monosaccharides by yeast present in soy sauce.^72^ Pinene peak (drift time, 9.68 ms) was observed in the ion mobility spectrum of the rosemary sprig (Figure 5D). Its identity was confirmed by recording a standard spectrum (Figure S5D). In fact, pinene is known to be present in rosemary plants, and its function is to inhibit bacterial activity.^73^ Peaks of carvone and farnesene (9.65 and 10.98 ms, respectively) were observed in the ion mobility spectrum of mint sprigs (Figure 5E). Their identities were confirmed by comparison of the specimen spectrum with the standard spectra (Figure S5E). The ion mobility spectrum of blue cheese features two prominent peaks at 10.43 and 11.47 ms (Figure 5F), which constitute the fingerprint of blue cheese. IMS analysis of the fabric exposed to incense smoke led to recording several peaks from 9.07 to 10.78 ms (Figure 5G). Gasoline revealed three distinct peaks at 8.85, 9.30, and 10.26 ms (Figure 5H).

Some of the real specimen VOCs have been verified using a corona discharge APCI Q-ToF mass spectrometer (Figures S6, S7, and Table S3). Please note that the analyzed matrices emit multiple volatile species. However, only some of them are present at a sufficiently high concentration, and have sufficiently high ionization efficiency in the R-APCI source, to be detected by the IMS instrument. Moreover, similar compounds (e.g., isomers) may not be distinguished. The putatively identified species—present in the real matrices—were subsequently verified by MS/MS (product ion scans). To confirm the presence of limonene in the tangerine peel, product ion scans were used for specific precursor ions (monomer: *m*/*z* 137.1330). As expected, the signal of limonene from tangerine peel was putatively matched with the limonene standard (Figure S8). Next, we performed an analysis of a nicotine patch. Ethyl acetate (*m*/*z* 89.0607) was found in the nicotine patch in the ion mobility spectrum (Figure 5B) and further characterized by a product ion scan (Figure S9), while nicotine (*m*/*z* 163.1236) was only detected in the nicotine patch by MS/MS (Figure S10). The dimer of ethanol (*m*/*z* 93.0905) was confirmed to be present in soy sauce by MS/MS (Figure S11). Additionally, the presence of pinene (*m*/*z* 137.1330) was confirmed in rosemary sprigs (Figure S12), while carvone (*m*/*z* 151.1119) (Figure S13) and farnesene (*m*/*z* 205.1950) (Figure S14) were identified in mint sprigs by MS/MS. Although the other matrices exhibited signals in Q-ToF mass spectra, these signals did not correspond to the signals observed in the ion mobility spectra. For instance, in the case of blue cheese, the Q-ToF-MS/MS scan revealed an intense peak of butyric acid (*m*/*z* 89.0608; Figure S15), which was expected (considering the presence of related compounds in *Penicillium*(74)) while there were no associated signals observed in the ion mobility spectrum (Figure 5F). It is known that the occurrence of butyric acid in blue cheese originates from microbial activity.^75^

**Figure 6:**
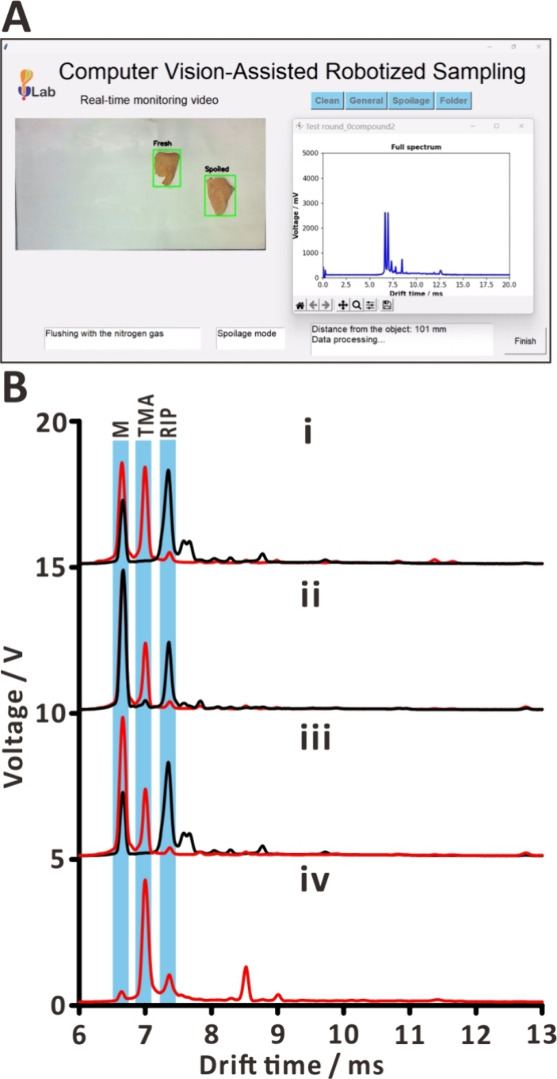
Discrimination between fresh and spoiled meat and spectra of food samples undergoing spoilage. (A) Screenshot of GUI for distinguishing food freshness. (B) Vapor analysis [(i) chicken breast, (ii) shrimp, (iii) salmon, and (iv) trimethylamine (10 μL of 4.4 × 10^–2^ M standard on the 15 mm filter paper disk)]. Lines: black–fresh meat; red–spoiled meat or standard. Labels: M—moisture; TMA—trimethylamine; RIP—reactant ion peak.

Another application of the presented autosampling platform is the classification of foodstuffs according to their freshness. The user needs to select the “Spoilage mode” in the analysis GUI and place a specimen in the drop-off zone. After the analysis, the corresponding spectrum and a labeled image of the food specimen are displayed in the GUI (Figure 6 and Movie S2). The specimen label—either “fresh” or “spoiled”—is based on the peak of trimethylamine in the ion mobility spectrum. In fact, trimethylamine is one of the major VOCs emitted during muscle food spoilage.^76,77^ It is one of the compounds in total volatile basic nitrogen, detected for evaluating freshness, and acts as a biomarker in the analysis of fish spoilage.^78−80^ If the trimethylamine signal (drift time, 7.04 ms) is higher than 1 V, the specimen is automatically labeled as “spoiled”. The CV-guided autosampling platform was able to classify multiple specimens in the same round. The GUI displays the picture of labeled fresh and spoiled meat specimens—present in the drop-off zone—along with the spectrum of the analyzed specimen (Figure 6A). We applied this method to distinguish between fresh and spoiled chicken breast, shrimp, and salmon stored in an incubator at 25 °C for 5 days. As expected, spoiled foodstuffs exhibited high-intensity peaks of trimethylamine in the ion mobility spectrum (Figure 6B). Notably, the increased intensity of a moisture-related peak (drift time, 6.67 ms) is attributed to the water released from the meat during spoilage.^81^ Using APCI-Q-ToF-MS (Figure S6)—operated in the MS and MS/MS modes—we could confirm the presence of trimethylamine in the vapor of spoiled chicken and shrimp but not salmon (Table S3 and Figure S16). However, all three specimens yielded trimethylamine signals in ion mobility spectra (Figure 6B). The lack of trimethylamine signals in the tandem mass spectrum of salmon can be due to the small amount emitted during the spoilage^82^ and the presence of other compounds with high proton affinity, which may suppress the trimethylamine signal. While this experiment demonstrated the simultaneous analysis of two specimens, we also verified the possibility of analyzing 10 specimens individually (Figure S17 and Movie S3).

## Conclusions

We have demonstrated an automated analytical platform for the rapid preparation-free qualitative analysis of solid specimens emitting VOCs. In this autosampling platform, the specimens are placed by the user in a random position within the specimen drop-off zone. The CV-guided robotic probe samples VOCs emanating from the specimen, and the gaseous extract is infused into the ion source of the ion mobility spectrometer. The autosampling platform features a GUI for seamless interaction. Various specimens were analyzed using this platform: tangerine peel, nicotine patch, soy sauce, rosemary sprig, mint sprig, fabric exposed to incense smoke, and gasoline. In one embodiment, the autosampling platform could automatically distinguish between fresh and spoiled meat. The current setup occupies little space, and it could readily be implemented for a vending machine style chemical analysis (cf. refs (38) and (83)). For example, chemical analysis of merchandise can be conducted by customers at markets and shopping centers. In the future, the autosampling platform can be upgraded by coupling the ion mobility separation stage with a mass analyzer to achieve selective analysis of individual VOCs and to facilitate analyte identification. It can also be adapted for the analysis of a greater number of randomly placed specimens.

## Supporting Information Available

Additional experimental details (system control; graphical user interfaces; operation of the autosampling platform); additional tables (S1–S3; optimization parameters; analytical performance; verification of the VOC signals); additional figures (S1–S21; pen-probe design; layout of the sampling system; photograph of the setup; recognition of the real specimens by the CV algorithm; spectra of real specimens and standards; verification of VOC signals recorded by IMS using APCI-Q-ToF-MS; full scan mass spectra; MS/MS scan mass spectra; simultaneous analysis; electronic control circuit; calibration GUI; evaluation of laser sensor accuracy; workflow of the analysis process); computer code (PDF)
Operation of the autosampling platform (MP4)
Distinguishing fresh and spoiled meat (MP4)
Analysis of 10 specimens (MP4)
