# Supplementary material for: Computer Vision-Assisted Robotized Sampling of Volatile Organic Compounds
Source: Anal Chem. 2024 Sep 26;96(41):16307–14. doi: 10.1021/acs.analchem.4c03361 (PMC11483429; doi:10.1021/acs.analchem.4c03361)
Supplement: Supplementary file 1 — ac4c03361_si_001.pdf [file ac4c03361_si_001.pdf]

SUPPORTING INFORMATION

Computer Vision-Assisted Robotized Sampling of Volatile Organic Compounds

Ching-Chi Chan, Noor Hidayat Abu Bakar, Chamarthi Maheswar Raju, Pawel L. Urban*

Department of Chemistry, National Tsing Hua University

101, Section 2, Kuang-Fu Rd., Hsinchu, 300044, Taiwan

* Corresponding author:

P.L. Urban (urban@mx.nthu.edu.tw)

Table of contents:

- additional experimental details;
- additional tables (S1-S3);
- additional figures (S1-S21);
- computer code;
- videos illustrating the operation of the auto-sampling platform (S1-S3) in separate files.

ADDITIONAL EXPERIMENTAL DETAILS

System control

The control unit took advantage of universal electronic modules following the recent trend in science. Prabhu, G. R. D.; Yang, T.-H.; Hsu, C.-Y.; Shih, C.-P.; Chang, C.-M.; Liao, P.-H.; Ni, H.-T.; Urban, P. L. Facilitating Chemical and Biochemical Experiments with Electronic Microcontrollers and Single-Board Computers. *Nat. Protoc.* **2020**, *15*, 925–990. ; Prabhu, G. R. D.; Urban, P. L. Elevating Chemistry Research with a Modern Electronics Toolkit. *Chem. Rev.* **2020**, *120*, 9482–9553. An Arduino Uno R3 (Arduino Uno; Centenary Material Company, Hsinchu, Taiwan) was connected to the relay to switch the solenoid valve on and off (**Figure S18**). Another Arduino board (Arduino Due; Centenary Material Company, Hsinchu, Taiwan) was used to capture spectral data from the IMS instrument. Four pins on the Arduino boards (2 pins on Arduino Uno R3 and 2 pins on Arduino Due) were connected to the 15-pin sub-D interface (port II) of the IMS instrument. The functions of the pins in this interface are: pin 3 – frameset signal; pin 4 – ground; pin 6 – triggering IMS instrument to collect data and save them in a .csv file; and pin 11 – analog signal. The frameset signal defines the time axis of the spectrum while the analog signal provides the spectral data. The pins 4 and 6 of the IMS instrument were connected to GND, and 13 pins in the Arduino Uno board, respectively. The pins 3 and 11 of the IMS instrument were connected to pins 5 and A0 on the Arduino Due board, respectively.

Graphical user interfaces

The whole experiment process is controlled by a program written in Python (version 3.11; Python Software Foundation, Fredericksburg, VA, USA). The Python program controls the movements of the robotic arm. The two Arduino programs are also triggered by the Python program to execute specific actions. To obtain the sample spectrum with the auto-sampling platform (**Figure 1A**), the Python program not only guides the robotic arm for sampling but also participates in object detection and determining whether there is any movement of objects within the specimen drop-off zone. The Python program displays two GUIs. The first GUI—called “calibration GUI” (**Figure S19**)—is used for calibrating the auto-sampling platform. The second GUI—called “analysis GUI” (**Figure 1B**)—is used for auto-sampling.

Operation of the auto-sampling platform

When the analysis GUI is activated, the solenoid valve opens to let the nitrogen gas flow to the pen-probe to remove possible contaminants. The webcam visualizes the drop-off zone to detect presence of the specimens. If no specimens are found, the system waits until the user places a specimen. The user can place real specimens or the filter paper disks (diameter, 15 mm) impregnated with a chemical standard in the specimen drop-off zone. In the case of the standard samples, the paper disks are placed on black trays (diameter, 6 cm), which are cleaned after each analysis. The Python program (with trained YOLOv5) identifies the specimen position and—upon confirming the absence of any movement in the dropoff zone—initiates a 5-s countdown timer. During the countdown notification, the user can place additional specimens in the specimen drop-off zone; however, this action disrupts the countdown. When an object is in motion in the drop-off zone, the dialogue box displays the message “Something is moving”. By monitoring the movement of an object, the auto-sampling platform not only monitors objects' entry into the specimen drop-off zone but also enables testing multiple specimens in

one row. After the countdown ends, the center coordinates of the detected object (pixels)—determined by the Python program—are converted into movement coordinates (*x*, *y*, *z*) – used for guiding the robotic arm.

The Python program starts analyzing the sample in the following sequence: (1) the robotic arm moves ~ 10 cm above the object position; (2) the calibrated laser sensor (*cf.* **Figure S20**) measures the distance between the sample and the holder of probe for 6 s while simultaneously hot nitrogen gas passes through the probe downwards to remove the VOCs present on the sample surface for 10 s ("blank time"); (3) the valve is closed, and the robotic arm goes down to ~ 1–3 mm above the specimen; (4) the pump starts aspirating VOCs into the IMS instrument and the IMS instrument starts recording spectra immediately for 15 s; (5) after sampling, the robotic arm moves back to its initial position, hot nitrogen gas is passed to clean the sample flow line, and the analysis GUI shows the full spectrum (average from 10-15 s) of the analyzed sample; (6) after completing the aforementioned process for one specimen within the specimen drop-off zone, the system can proceed to analysis of the next specimen. Once all the specimens present within the specimen drop-off zone have been analyzed, the Python program initiates the next round of object detection while waiting for the placement of new specimens. If the specimen that has been sampled is still present, the analysis GUI displays the message "Please remove the object". The workflow of the auto-sampling platform is shown in **Figure S21**.

ADDITIONAL TABLES

Table S1. Optimization parameters, default values, optimization ranges, and selected values.

Parameter	Default	Range	Selected value
distance between the bottom of the pen-probe and the sample	2 mm	2-10 mm	2 mm
flow rate of the auto-sampling platform	48 mL min^{-1}	6-48 mL min^{-1}	48 mL min^{-1}
drift tube temperature	80 °C	20-90 °C	80 °C
heated nitrogen gas temperature	40 °C	22-100 °C	100 °C
sampling time	20 s	10-30 s	20 s
blank time	10 s	5-25 s	10 s

Table S2. Analytical performance of the CV-guided automated sampling system. The LODs and LOQs were calculated based on the equations: LOD = 3.3 × (*Sy*/*S*), LOQ = 10 × (*Sy*/*S*), where *Sy* represents the standard deviation of *y*-intercepts of regression lines and *S* represents the slope of the calibration curve. Determination of LODs (Limits of Detection) and LOQs (Limit of Quantification). https://arts-sciences.und.edu/academics/chemistry/kubatova-research-group/_files/docs/determination_of_lods_new.pdf (accessed 2024–6–20).

Compound name	Formula	Drift time / ms	Calibration equation (U / mV; $C_{surface}$ / mol m^{-2})	R^2	LOD / mol m^{-2}	LOQ / mol m^{-2}	Repeatability (RSD) / % (n = 10)	Reproducibility (RSD) / % (n = 6; 6 out of 8 days; each day, n = 3)
ethyl acetate	$C_4H_8O_2$	10.06	$U = (2.08 \times 10^6 \pm 1.63 \times 10^6)C_{surface} + (47.68 \pm 20.45)$	0.9814	3.28×10^{-5}	9.94×10^{-5}	12.4	9.7
limonene	$C_{10}H_{16}$	8.97	$U = (8.57 \times 10^3 \pm 0.67 \times 10^3)C_{surface} + (379.21 \pm 17.05)$	0.9818	6.57×10^{-3}	1.99×10^{-2}	10.2	12.1
pinene	$C_{10}H_{16}$	10.46	$U = (5.22 \times 10^3 \pm 0.69 \times 10^3)C_{surface} + (244.08 \pm 17.31)$	0.9497	1.10×10^{-2}	3.32×10^{-2}	17.0	14.0
trimethylamine	C_3H_9N	7.04	$U = (2.10 \times 10^6 \pm 0.12 \times 10^6)C_{surface} + (-133.41 \pm 75.98)$	0.9900	1.19×10^{-4}	3.61×10^{-4}	5.9	6.5

Table S3. Verification of the VOC signals—recorded by IMS—in real specimens using APCI-Q-ToF-MS.

Real sample	Putative compound	Formula	Exact mass (*m/z*)	Accurate mass (*m/z*)	Mass error (ppm)
tangerine peel	limonene	$C_{10}H_{16}$	137.1329	137.1330	0.7
nicotine patch	ethyl acetate	$C_4H_8O_2$	89.0602	89.0607	5.6
nicotine patch	nicotine	$C_{10}H_{14}N_2$	163.1234	163.1236	1.2
soy sauce	ethanol	C_2H_6O	47.0496	47.0497	2.1
rosemary sprig	pinene	$C_{10}H_{16}$	137.1329	137.1330	0.7
mint sprig	carvone	$C_{10}H_{14}O$	151.1122	151.1119	1.9
mint sprig	farnesene	$C_{15}H_{24}$	205.1956	205.1950	2.9
blue cheese	butyric acid	$C_4H_8O_2$	89.0602	89.0608	6.7
spoiled chicken	trimethylamine	C_3H_9N	60.0812	60.0810	3.3
spoiled shrimp	trimethylamine	C_3H_9N	60.0812	60.0810	3.3

ADDITIONAL FIGURES

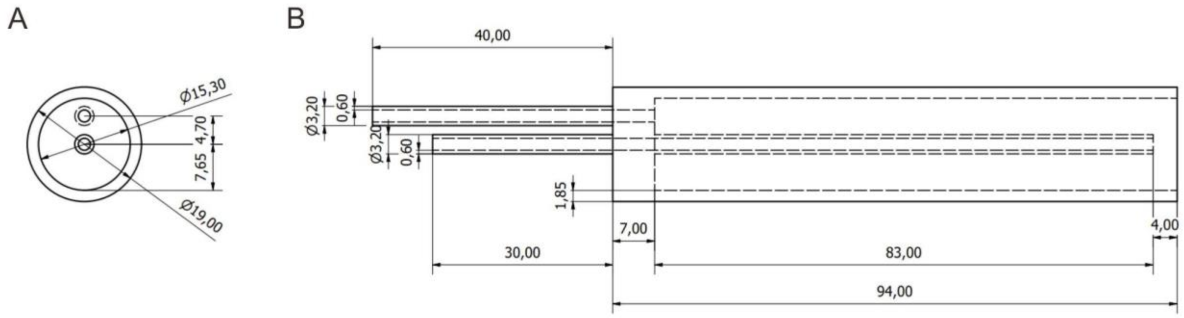

Figure S1. Pen-probe design: (A) top view; (B) side view. Dimension unit: mm.

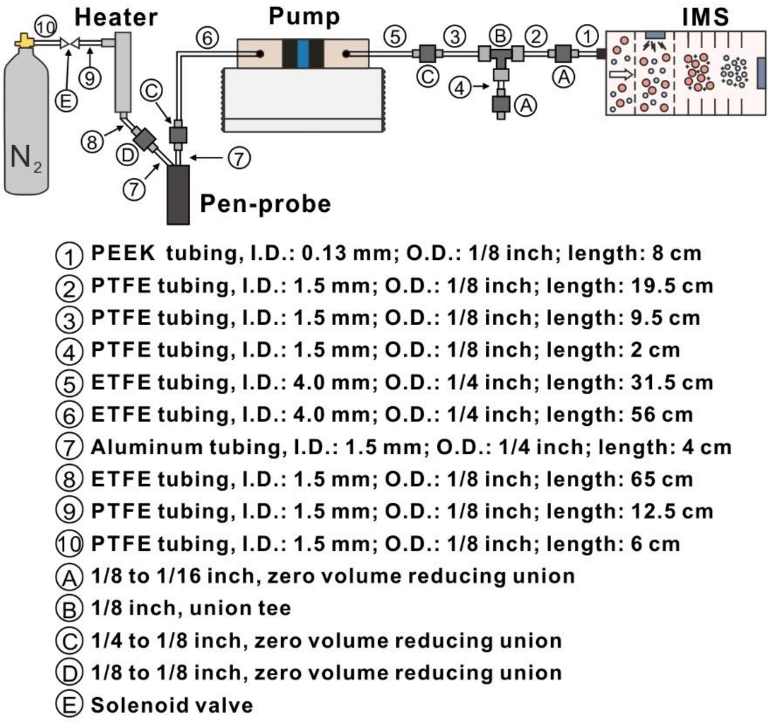

Figure S2. Layout of the sampling system.

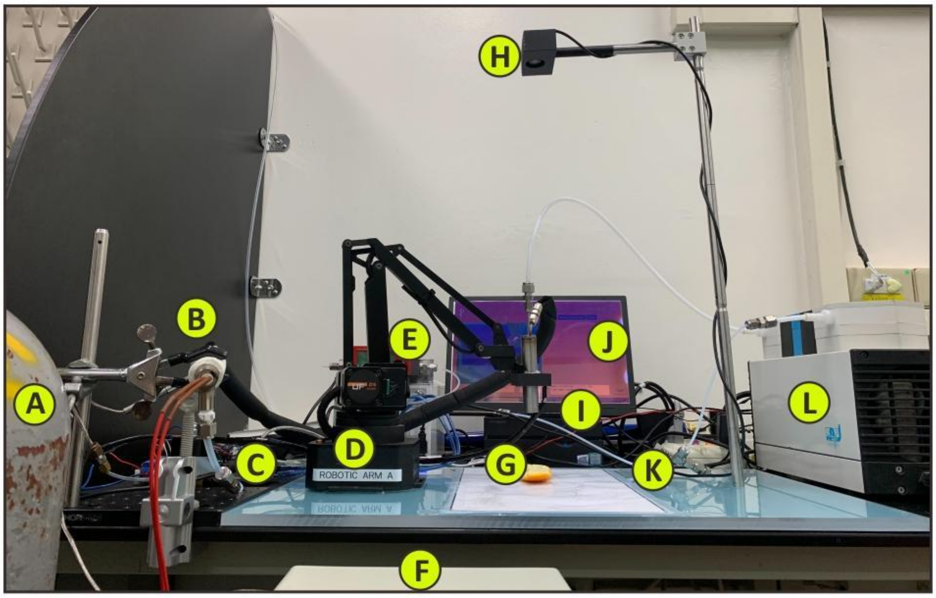

Figure S3. Photograph of the setup. (A) nitrogen cylinder gas; (B) heater; (C) control unit; (D) robotic arm; (E) IMS instrument; (F) heater controller; (G) specimen; (H) webcam; (I) pen-probe; (J) computer; (K) specimen drop-off zone on acrylic board; (L) pump.

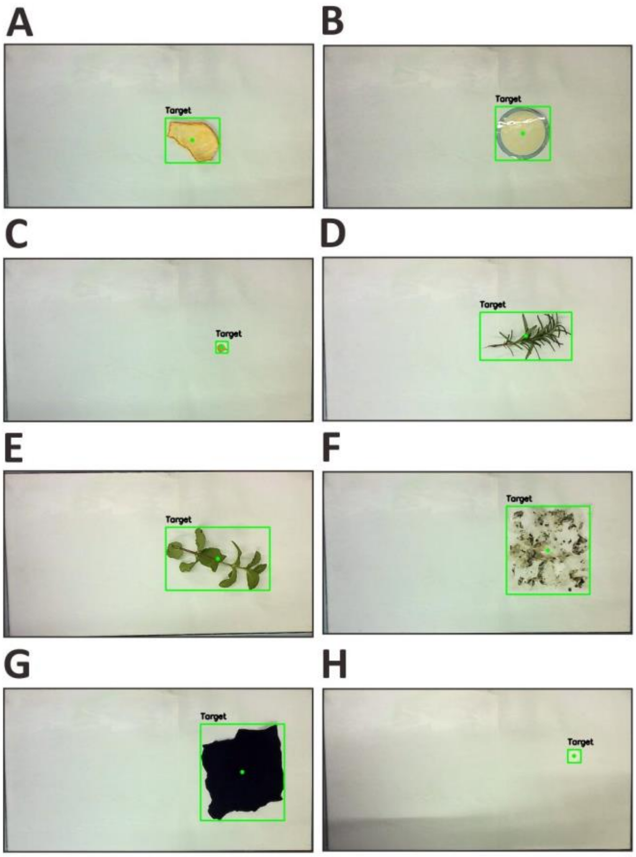

Figure S4. Recognition of the real specimens by the CV algorithm: (A) tangerine peel; (B) nicotine patch; (C)10 μL of soy sauce on the filter paper disk; (D) rosemary sprig; (E) mint sprig; (F) blue cheese; (G) fabric exposed to incense smoke; and (H) 10 μL of gasoline on the filter paper disk.

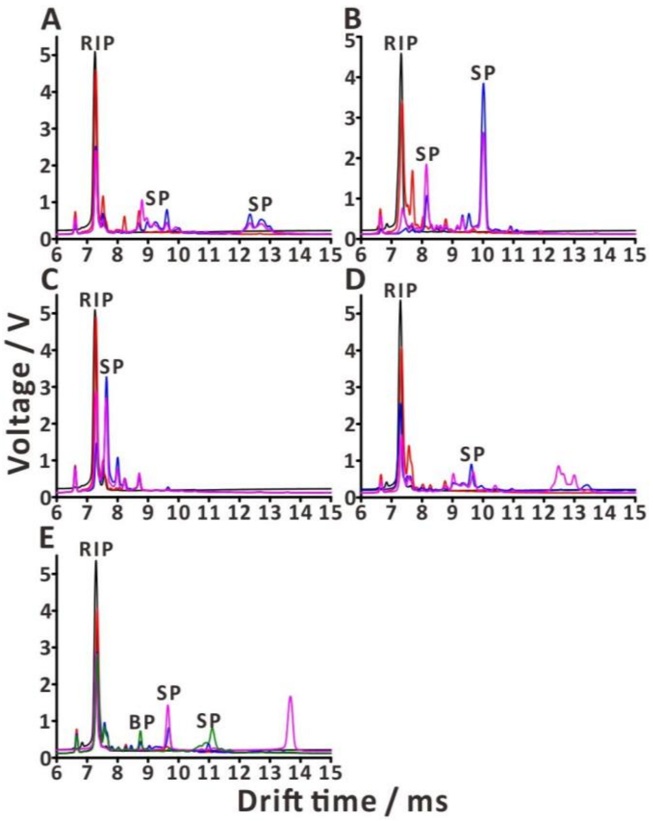

Figure S5. Spectra of real specimens and standards: (A) tangerine peel, limonene; (B) nicotine patch, ethyl acetate; (C) soy sauce, ethanol; (D) rosemary sprig, pinene; and (E) mint sprig, carvone (purple), and farnesene (green). The standards correspond to the peaks recorded in the spectra of the real specimens. A 15 mm filter paper disk was prepared with 2 μL each of pure limonene, pinene, carvone, and farnesene, as well as 10 μL of 10^{-1} M ethyl acetate (in water) and ethanol (in water). Lines: black – instrumental blank; red – system blank; blue – real specimen; purple and green – standards. Labels: RIP – reactant ion peak; BP – blank peak; SP – sample peak.

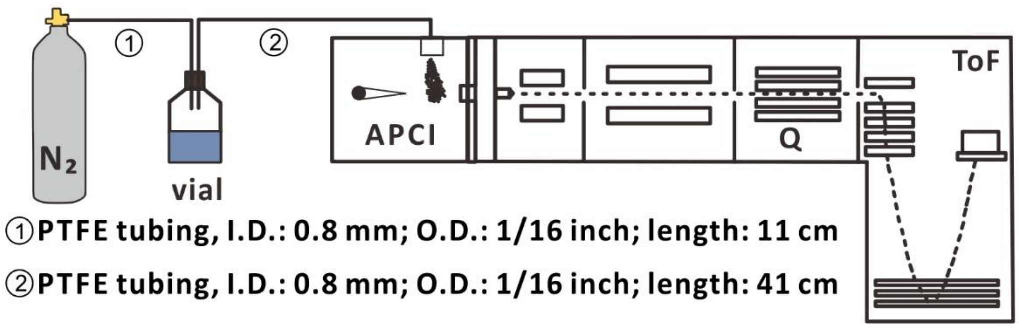

Figure S6. Verification of VOC signals recorded by IMS using APCI-Q-ToF-MS. Parameters: nebulizing gas flow rate, 3 L min^{-1}; drying gas, 5 L min^{-1}; desolvation line temperature, 250 °C; heated block temperature, 250 °C; desolvation line temperature, 250 °C; corona needle voltage, 4.5 kV; nitrogen gas pressure for headspace flushing, 5 psi, analysis time, 1 min.

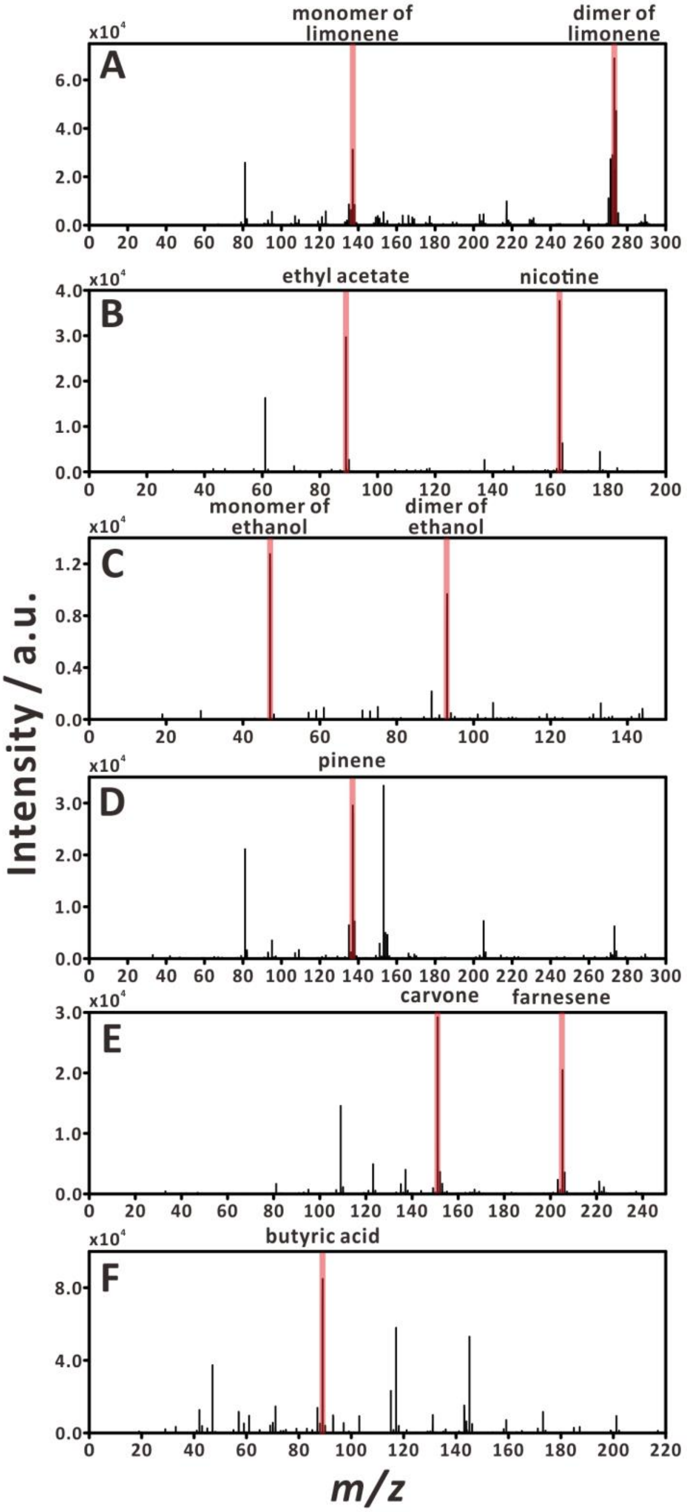

Figure S7. Full scan mass spectra of the vapors sampled from the real specimens: (A) tangerine peel; (B) nicotine; (C) soy sauce; (D) rosemary sprigs; (E) mint sprigs; (F) blue cheese.

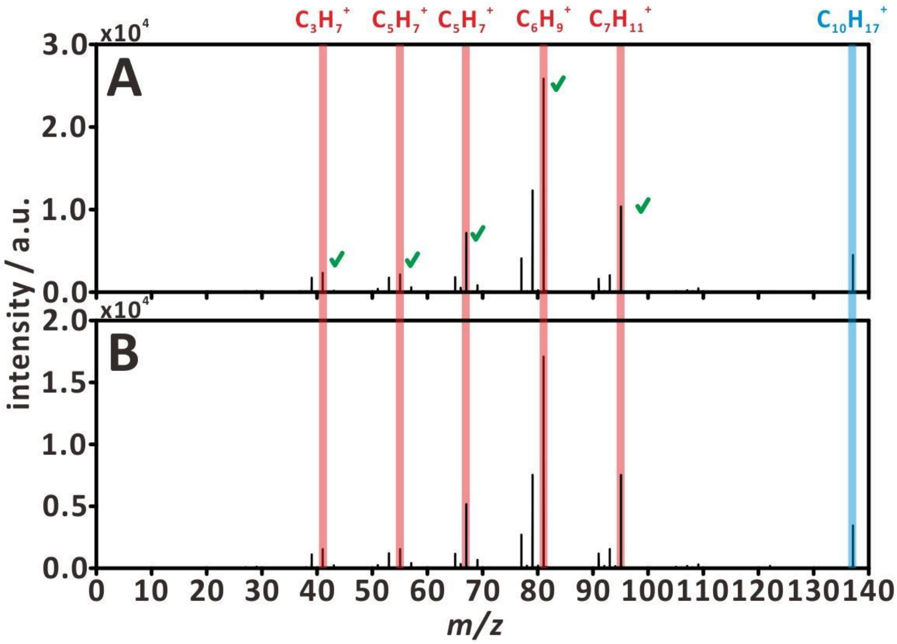

Figure S8. MS/MS scan mass spectra of tangerine peel and limonene standard: (A) tangerine peel in vial; (B) 5 mL of 10^{-2} M limonene (in methanol) in vial. Tangerine peel and limonene standard were sampled, and their ions were fragmented at the collision voltages from -8 V to -42 V. Precursor ion *m/z*: 137.1330. Spectra were averaged from 1 min record. Symbols: green tick – good match.

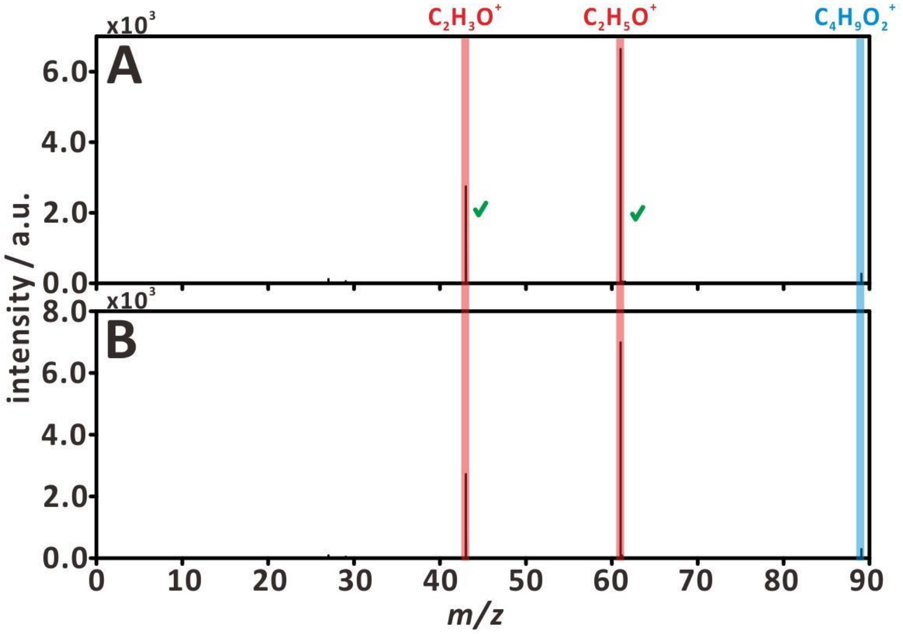

Figure S9. MS/MS scan mass spectra of nicotine patch and ethyl acetate standard: (A) nicotine patch in vial; (B) 5 mL of 10^{-4} M ethyl acetate (in water) in vial. Nicotine patch and ethyl acetate standard were sampled, and their ions were fragmented at the collision voltages from -8 V to -42 V. Precursor ion *m/z*: 89.0607. Spectra were averaged from 1 min record. Symbols: green tick – good match.

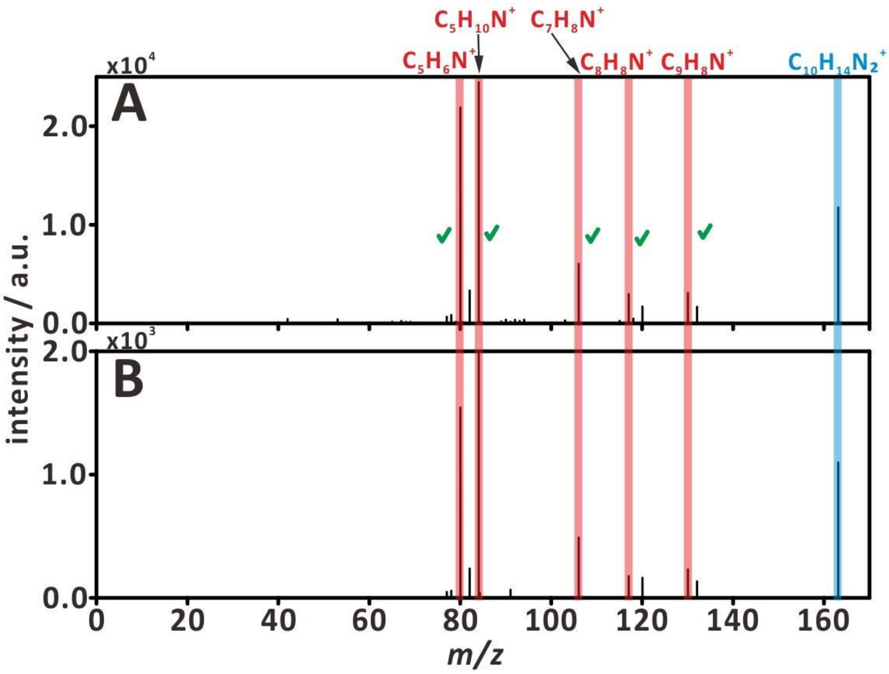

Figure S10. MS/MS scan mass spectra of nicotine patch and nicotine standard: (A) nicotine patch in vial; (B) 5 mL of 10^{-3} M nicotine (in water) in vial. Nicotine patch and nicotine standard were sampled, and their ions were fragmented at the collision voltages from -8 V to -42 V. Precursor ion *m/z*: 163.1236. Spectra were averaged from 1 min record. Symbols: green tick – good match.

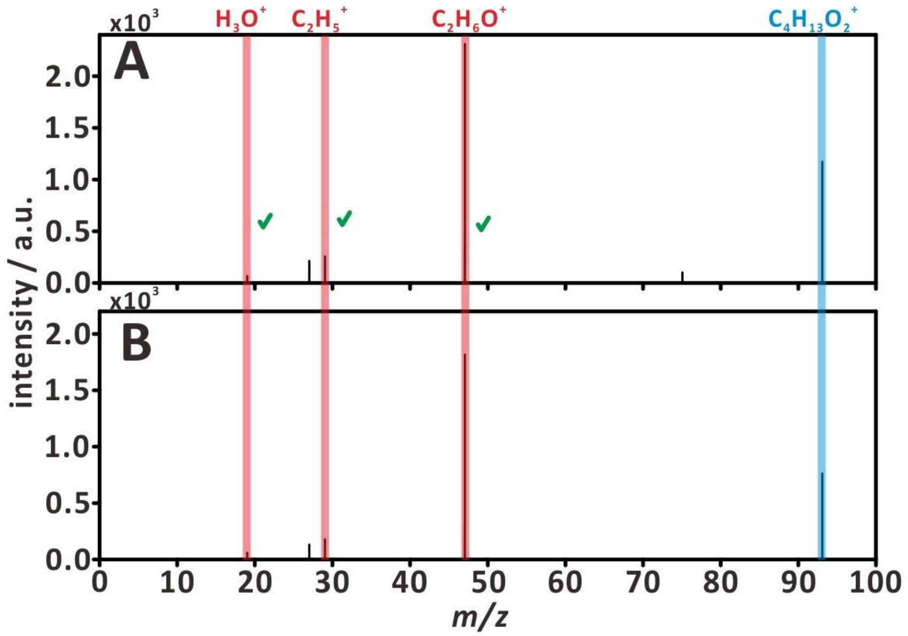

Figure S11. MS/MS scan mass spectra of soy sauce and ethanol standard: (A) 2 mL of soy sauce in vial; (B) 5 mL of 10^{-2} M ethanol (in water) in vial. Soy sauce and ethanol standard were sampled, and their ions were fragmented at the collision voltages from -8 V to -42 V. Precursor ion (ethanol dimer) *m/z*: 93.0905. Spectra were averaged from 1 min record. Symbols: green tick – good match.

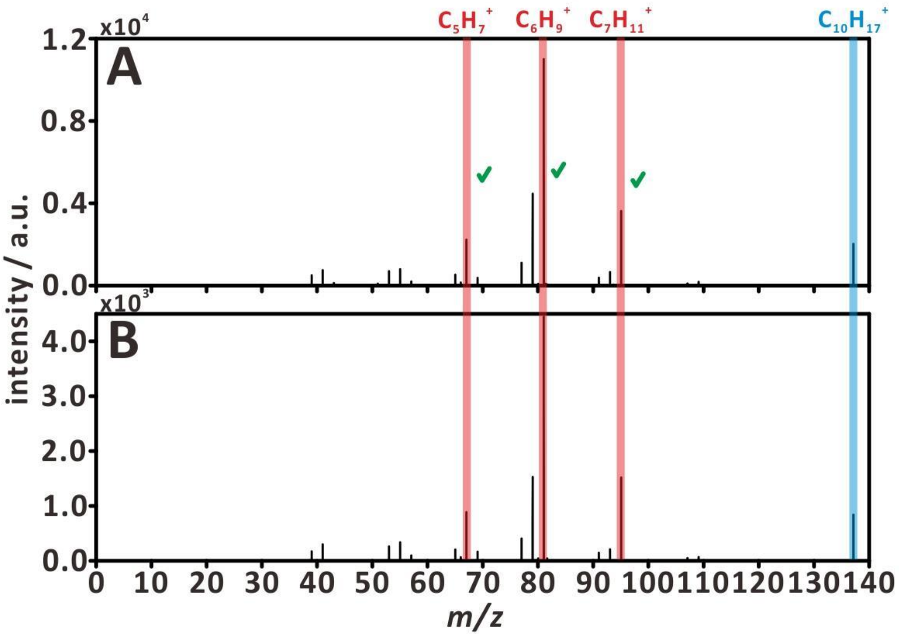

Figure S12. MS/MS scan mass spectra of rosemary sprig and pinene standard: (A) rosemary sprig in vial; (B) 5 mL of 10^{-2} M pinene (in methanol) in vial. Rosemary sprig and pinene standard were sampled, and their ions were fragmented at the collision voltages from -8 V to -42 V. Precursor ion *m/z*: 137.1330. Spectra were averaged from 1 min record. Symbols: green tick – good match.

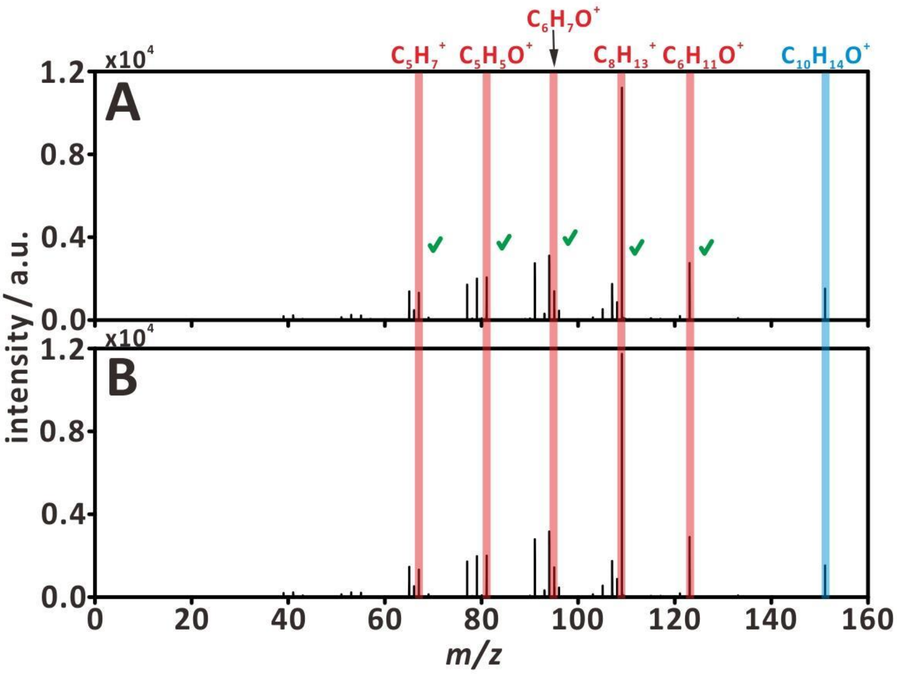

Figure S13. MS/MS scan mass spectra of mint sprig and carvone standard: (A) mint sprig in vial; (B) 5 mL of 10^{-3} M carvone (in methanol) in vial. Mint sprig and carvone standard were sampled, and their ions were fragmented at the collision voltages from -8 V to -42 V. Precursor ion *m/z*: 151.1119. Spectra were averaged from 1 min record. Symbols: green tick – good match.

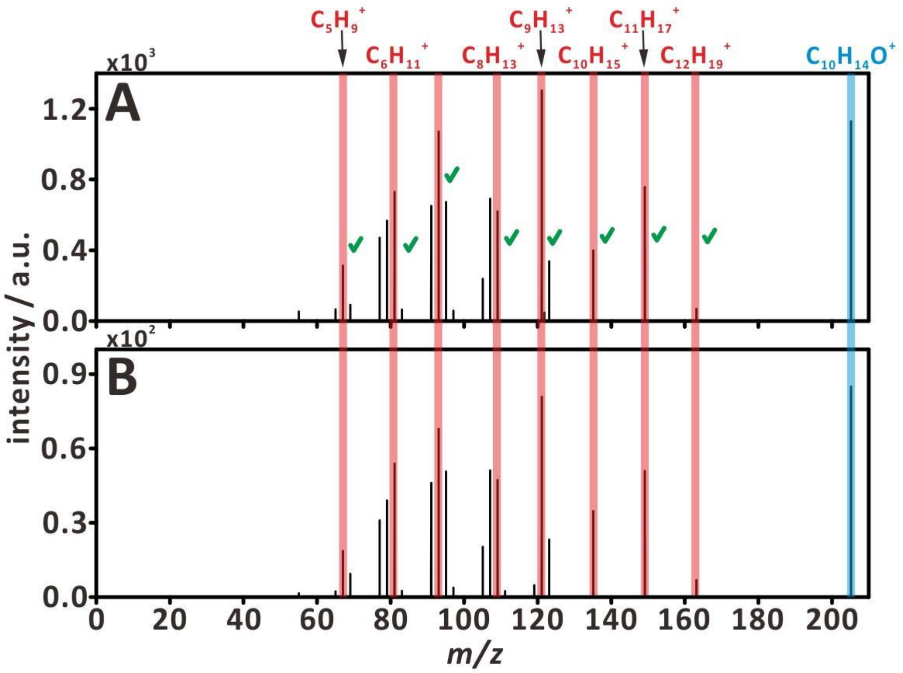

Figure S14. MS/MS scan mass spectra of mint sprig and farnesene standard: (A) mint sprig in vial; (B) 5 mL of 10^{-3} M farnesene (in methanol) in vial. Mint sprig and farnesene standard were sampled, and their ions were fragmented at the collision voltages from -8 V to -42 V. Precursor ion *m/z*: 205.1950. Spectra were averaged from 1 min record. Symbols: green tick – good match.

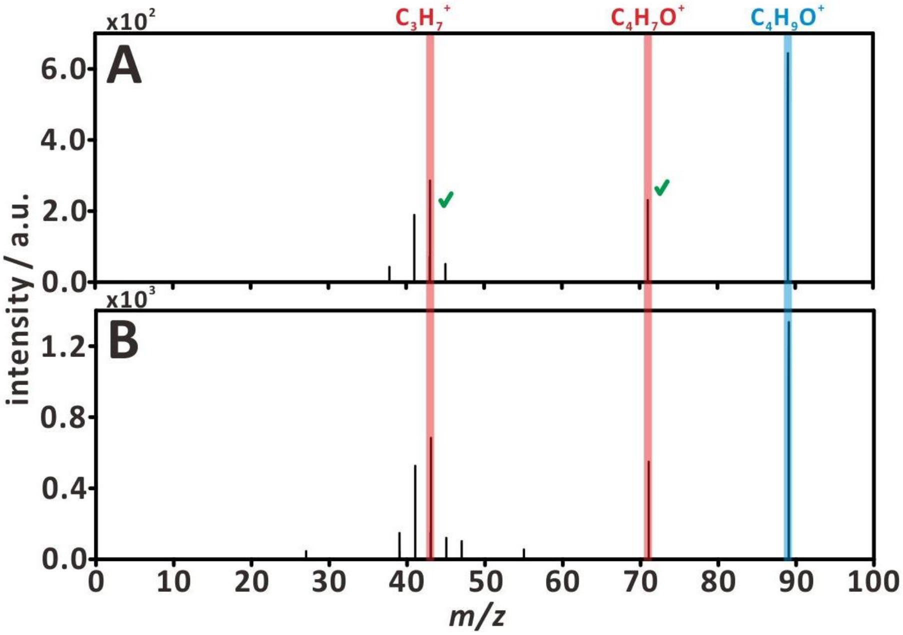

Figure S15. MS/MS scan mass spectra of blue cheese and butyric acid standard: (A) blue cheese in vial; (B) 5 mL of 10^{-4} M butyric acid (in water) in vial. Blue cheese and butyric acid standard were sampled, and their ions were fragmented at the collision voltages from -8 V to -42 V. Precursor ion *m/z*: 89.0608. Spectra were averaged from 1 min record. Symbols: green tick – good match.

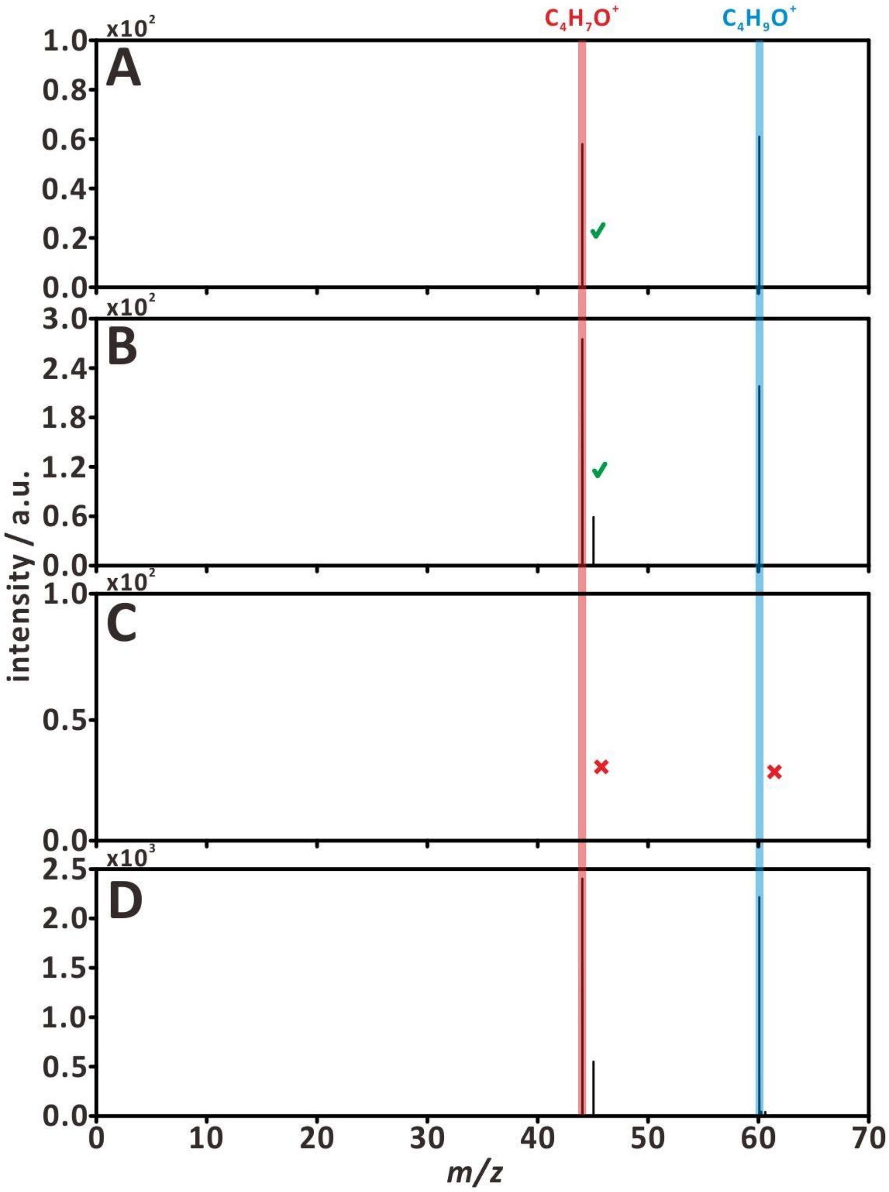

Figure S16. MS/MS scan mass spectra of spoiled chicken, shrimp, and trimethylamine standard: (A) spoiled chicken in vial; (B) spoiled shrimp in vial; (C) spoiled salmon in vial; (D) 5 mL of 4.4×10^{-3} M trimethylamine (in water) in vial. Spoiled chicken, spoiled shrimp, spoiled salmon, and trimethylamine standard were sampled, and their ions were fragmented at the collision voltages from -8 V to -42 V. Precursor ion *m/z*: 60.0810. Spectra were averaged from 1 min record. Symbols: green tick – good match; red cross – mismatch.

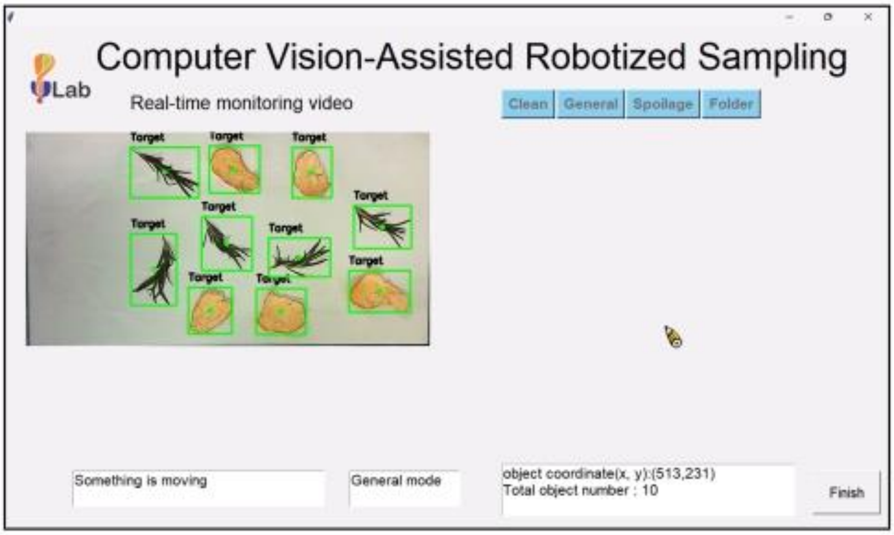

Figure S17. Demonstration of simultaneous analysis of 10 specimens (snapshot from **Movie S3**). The specimens were randomly placed on the drop-off zone at a time to let the device perform automatic analysis.

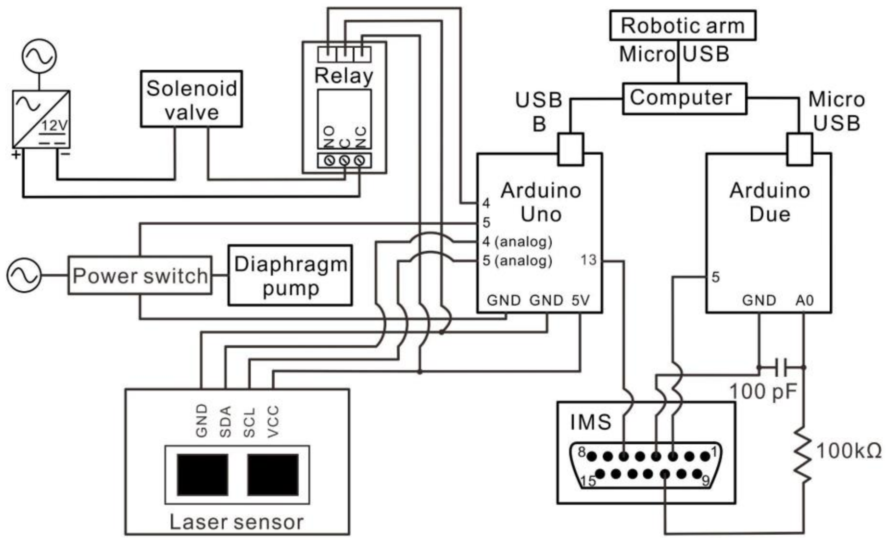

Figure S18. Electronic control circuit of the sampling system.

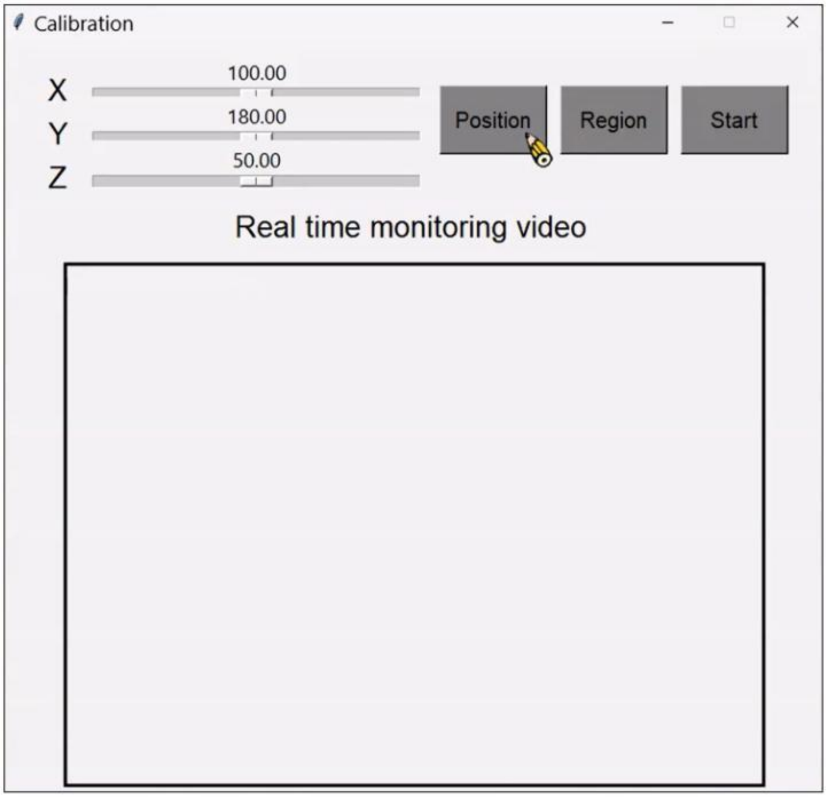

Figure S19. Screenshot of the calibration GUI.

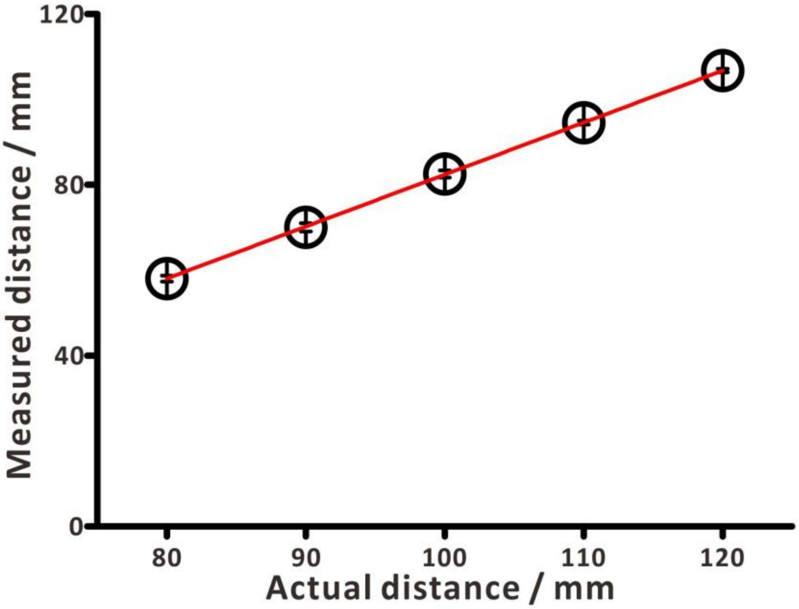

Figure S20. Evaluation of laser sensor accuracy. Measured distance refers to the distance between the laser and the wall, as determined by the laser sensor. Actual distance refers to the distance between the laser and the wall, as measured by the ruler.

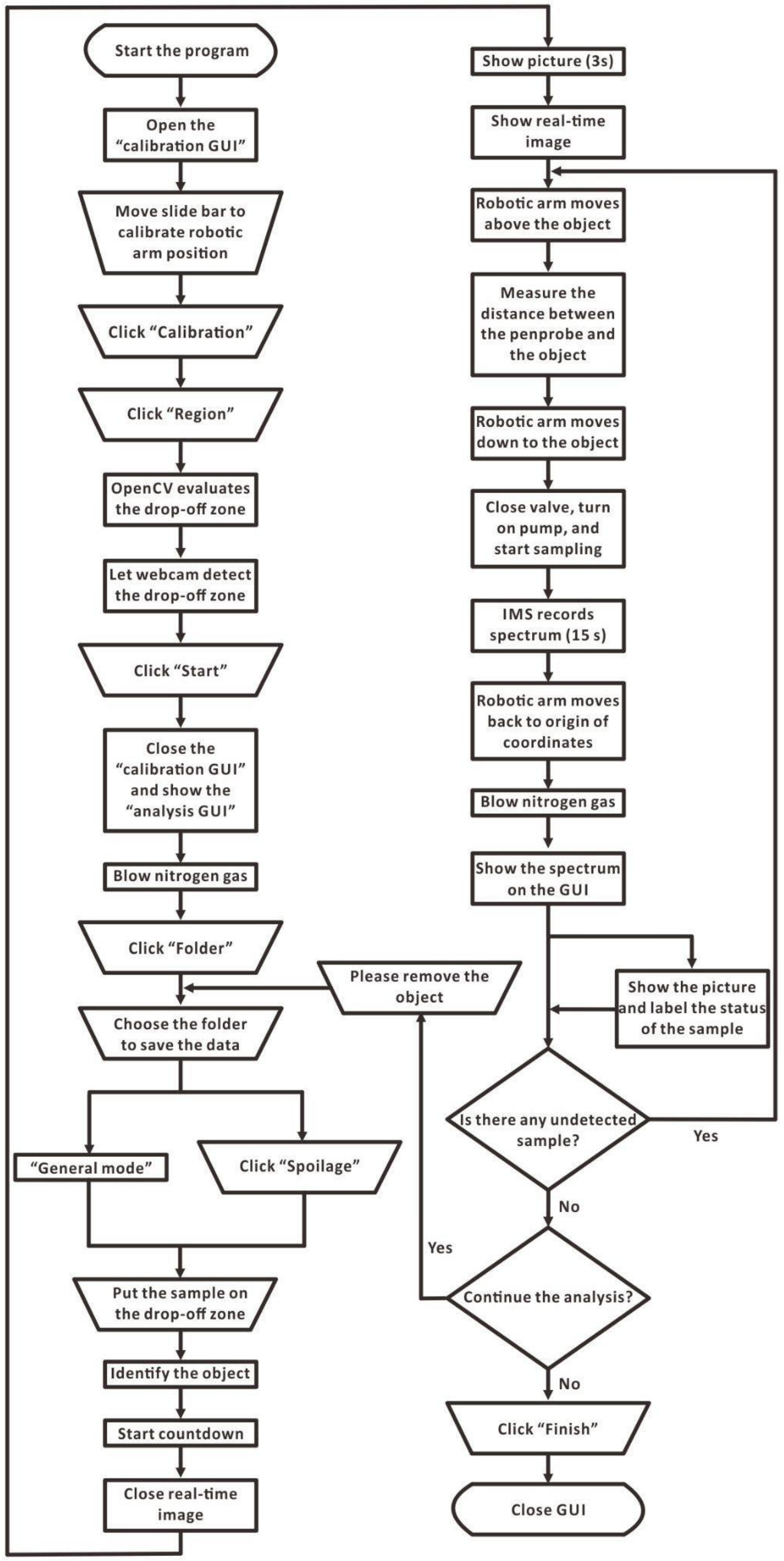

Figure S21. Workflow of the analysis process.

COMPUTER CODE

Arduino Uno (control of the sampling system)

```
#include "Adafruit_VL53L1X.h"  //laser sensor library
#define IRQ_PIN 2
#define XSHUT_PIN 3

Adafruit_VL53L1X vl53 = Adafruit_VL53L1X(XSHUT_PIN, IRQ_PIN);
int detectionduration = 5000;

void setup() {
  Serial.begin(9600); // Setup serial connection for print out to console
  pinMode(5, OUTPUT); // Pump
  pinMode(4, OUTPUT); // Valve
  pinMode(13, OUTPUT); // IMS
  digitalWrite(13,HIGH);
  while (!Serial) delay(10);
  Wire.begin();
  if (! vl53.begin(0x29, &Wire)) {
    while (1)       delay(10);
  }
  if (! vl53.startRanging()) {
    while (1)       delay(10);
  }
  vl53.setTimingBudget(50);

}
void loop() {
  if (Serial.available ()>0){
  int inByte = Serial.read();
  switch (inByte) {

  case '1': //valve open
  digitalWrite(4,HIGH);
  break;

  case'2'://valve close
  digitalWrite(4,LOW);
  break;

  case'3'://trigger IMS
  digitalWrite(13,LOW);
  delay(20000);
  digitalWrite(13,HIGH);
  break;

  case'4'://turn ON pump
  digitalWrite(5,HIGH);
  break;
```

```
  case'5'://turn OFF pump
  digitalWrite(5,LOW);
  break;

  case'6'://measure the distance
  int16_t distance;
  unsigned long starttime = millis();
  while (millis()-starttime < detectionduration){
    // new measurement for the taking
    distance = vl53.distance();
    Serial.println(distance);
    vl53.clearInterrupt();
  }
  }
  }

}
```

Arduino Due (obtaining the spectrum information)

```
int triggerpin = 5; //frameset signal
int digitalpin = A0;  //analog signal
int p = 0;
int samplingduration = 5000;
unsigned long x[3200][1];
float h[3200][2];

void setup() {
  Serial.begin(115200);
  pinMode(digitalpin, INPUT);
  pinMode(triggerpin, INPUT);
}

void loop() {
  if (Serial.available()>0){
    int inbyte=Serial.read();
    switch (inbyte){
    case '1':
      p = 0;
      while (p < 1) {
        if (digitalRead(triggerpin) == 1) { // Receive frameset signal from
IMS
          for (int s = 0; s < 3200; s++) { // Record the first spectrum
            x[s][0] = micros();
            h[s][0] = analogRead(A0);
          }
          p = p + 1;
        }
      }

      while (p < 10) { // Record 10 spectra
        if (digitalRead(triggerpin) == 1) {
          for (int s = 0; s < 3200; s++) {
            x[s][0] = micros();
            h[s][1] = analogRead(A0);
          }
          p = p + 1;
          for (int s = 0; s < 3200; s++) {
            h[s][0] = h[s][0] + h[s][1];
          }
        }
      }

      for (int s = 0; s < 3200; s++) { // Average 50 spectra
        h[s][0] = h[s][0] / p;
      }

      for (int s = 0; s < 3200; s++) { // Report the drift time and the
intensity

        Serial.print(x[s][0] - x[0][0]);
```

```
        Serial.print(",");
        Serial.print(h[s][0]);
        Serial.print("\n");
        delay(0);
      }

      break;

      case '2':
      p = 0;
      while (p < 1) {
        if (digitalRead(triggerpin) == 1) { // Receive frameset signal from
IMS
          for (int s = 0; s < 3200; s++) { // Record the first spectrum
            x[s][0] = micros();
            h[s][0] = analogRead(A0);
          }
          p = p + 1;
        }
      }

      unsigned long starttime = millis();
      while (millis()-starttime < samplingduration) { // Record spectra for
5 seconds

        if (digitalRead(triggerpin) == 1) {
          for (int s = 0; s < 3200; s++) {
            x[s][0] = micros();
            h[s][1] = analogRead(A0);
          }
          p = p + 1;
          for (int s = 0; s < 3200; s++) {
            h[s][0] = h[s][0] + h[s][1];
          }
        }
      }

      for (int s = 0; s < 3200; s++) { // Average the spectra
        h[s][0] = h[s][0] / p;
      }

      for (int s = 0; s < 3200; s++) { // Report the drift time and the
intensity

        Serial.print(x[s][0] - x[0][0]);
        Serial.print(",");
        Serial.print(h[s][0]);
        Serial.print("\n");
        delay(0);
      }
      break;
}
}
```

```
}
```

Python code (controlling the entire analysis)

```
import cv2  # Process the stream video
import numpy as np
import tkinter as tk    # Create the graphical user interface
from tkinter import filedialog as fd
from PIL import Image, ImageTk
import time
import threading    # Enable work the function at the same time
import os
import sys
sys.path.append(os.path.join(os.path.dirname(__file__),'..'))
from uarm.wrapper import SwiftAPI   # uArm Swift Pro library
import serial   # Connect with Arduino boards
import matplotlib   # Show the spectrum
import matplotlib.pyplot as plt
import matplotlib.font_manager as fm
import shutil   # Transfer the file
import yolov5   # Machine learning for object detection

swift = SwiftAPI(port='COM13')  # Connect to uArm Swift Pro
ser = serial.Serial('COM5', 9600)   # Connect to Arduino Uno
arduino = serial.Serial('COM7', 115200, timeout=1)  # Connect to Arduino
Due
swift.set_position(x=100,y=180,z=50,speed=75)
time.sleep(1)

model = yolov5.load('yolov5s.pt')   # Used the simplest model in the yolo
# set model parameters
model.conf = 0.002  # NMS confidence threshold
model.iou = 0  # NMS IoU threshold
model.agnostic = True  # NMS class-agnostic
model.multi_label = False  # NMS multiple labels per box
model.max_det = 10  # maximum number of detections per image

left = 0
right = 0
top = 0
bottom = 0
calibration_in_progress = False
RIP_value = None

class Caliexperiment:
    def __init__(self, window, window_title):
        self.win = window
        self.win.resizable(False, False)
        self.win.geometry('750x690+265+20')
        self.win.title(window_title)

        self.video_source = 0
        self.cap = cv2.VideoCapture(self.video_source)
        self.user_input = None
```

```
        self.canvas = tk.Canvas(window, width=self.cap.get(3), 
height=self.cap.get(4))
        self.canvas.create_rectangle(3, 3, 640, 480, width = 3)
        self.canvas.place(x=55, y=200)

        self.text = tk.Label(window, text="Real time monitoring video")
        self.text.config(font='Arial 20')
        self.text.place(x=210, y=150)

        self.posi = tk.Button(text='Position', background='gray', 
fg='black', command=self.position)
        self.posi.config(width=8, heigh=2, font='Arial 15')
        self.posi.place(x=400, y=40)

        self.cali = tk.Button(text='Region', background='gray')
        self.cali.config(width=8, height=2, font='Arial 15', 
command=self.calibrate)
        self.cali.place(x=510, y=40)

        self.btn = tk.Button(text='Start', background='gray')
        self.btn.config(width=8, height=2, font='Arial 15', 
command=self.close_window)
        self.btn.place(x=620, y=40)

        self.X_lb = tk.Label(fg='black', text='X')
        self.X_lb.config(font='Arial 20')
        self.X_lb.place(x=40, y=25)

        self.Y_lb = tk.Label(fg='black', text='Y')
        self.Y_lb.config(font='Arial 20')
        self.Y_lb.place(x=40, y=65)

        self.Z_lb = tk.Label(fg='black', text='Z')
        self.Z_lb.config(font='Arial 20')
        self.Z_lb.place(x=40, y=105)

        self.X_s = tk.Scale(orient="horizontal", width=10, length=300)
        self.X_s.config(from_=50, to=150, resolution=0.01, digits=5, 
command=self.change_X_cal)
        self.X_s.set(100)
        self.X_s.place(x=80, y=10)

        self.Y_s = tk.Scale(orient="horizontal", width=10, length=300)
        self.Y_s.config(from_=130, to=230, resolution=0.01, digits=5, 
command=self.change_Y_cal)
        self.Y_s.set(180)
        self.Y_s.place(x=80, y=50)

        self.Z_s = tk.Scale(orient="horizontal", width=10, length=300)
        self.Z_s.config(from_=0, to=100, resolution=0.01, digits=5, 
command=self.change_Z_cal)
        self.Z_s.set(50)
        self.Z_s.place(x=80, y=90)
```

```
        self.update_delay = 10  # Set the OpenCV screen update interval
(milliseconds)
        self.update()

        self.win.mainloop()

    def change_X_cal(self,value):
        swift.set_position(x=float(value))

    def change_Y_cal(self,value):
        swift.set_position(y=float(value))

    def change_Z_cal(self,value):
        swift.set_position(z=float(value))

    def position(self):
        swift.set_position(z=100)

    def update(self):
        global calibration_in_progress
        ret, frame = self.cap.read()
        if calibration_in_progress and ret:
            frame_gray = cv2.cvtColor(frame, cv2.COLOR_BGR2GRAY)
            frame_gray = cv2.GaussianBlur(frame_gray, (11, 11), 0)
            canny = cv2.Canny(frame_gray, 100, 150)
            contours, hierarchy = cv2.findContours(canny,
cv2.RETR_EXTERNAL, cv2.CHAIN_APPROX_NONE)
            for cnt in contours:
                cv2.drawContours(frame, [cnt], -1, (255, 0, 0), 4)
                area = cv2.contourArea(cnt)
                if area > 5000:
                    length = cv2.arcLength(cnt, True)
                    vertices = cv2.approxPolyDP(cnt, length * 0.02, True)
                    x, y, w, h = cv2.boundingRect(cnt)
                    object_x = x + w // 2
                    object_y = y + h // 2
                    cv2.rectangle(frame, (x, y), (x + w, y + h), (0, 255,
0), 2)
                    cv2.circle(frame, (object_x, object_y), 4, (0, 255, 0),
-1)
                    global left, right, top, bottom
                    left = np.min(vertices[:, 0, 0])
                    right = np.max(vertices[:, 0, 0])
                    top = np.min(vertices[:, 0, 1])
                    bottom = np.max(vertices[:, 0, 1])

            self.photo =
ImageTk.PhotoImage(image=Image.fromarray(cv2.cvtColor(frame,
cv2.COLOR_BGR2RGB)))
            # The function of Image.fromarray is to convert OpenCV that has
converted the screen color into an image object of PIL
            # ImageTk.PhotoImage() for convert PIL image objects to image
```

```
formats supported by Tkinter
            self.canvas.create_image(0, 0, image=self.photo, anchor=tk.NW)
            # canvas.create_image used for add the image in Canvas
        self.win.after(self.update_delay, self.update)

    def calibrate(self):
        global calibration_in_progress,RIP_value
        max_intensity = 0
        max_ms = 0
        x_axis = np.zeros(3200)
        y_axis = np.zeros(3200)
        arduino.write(b'1')
        k = 0
        while k < 3200:
            b = arduino.readline()
            b = b.decode()
            b = b.strip()
            values = b.split(",")
            if len(values) == 2:
                x_axis[k] = float(values[0])
                y_axis[k] = float(values[1])
                k += 1

        # Get the RIP drift time and its intensity
        max_intensity = np.argmax(y_axis)
        print(x_axis[max_intensity])
        max = np.max(y_axis)
        max_ms = float(int(x_axis[max_intensity]/10) / 100.0)
        RIP_value = max_ms + 0.07
        print("RIP ms: " + str(RIP_value) + " ms")
        print("IMS RIP intensity: " + str(round((5 * max / 1023), 2)) +"
V")
        calibration_in_progress = True

    def close_window(self):
        global calibration_in_progress
        calibration_in_progress = False
        self.cap.release()
        cv2.destroyAllWindows()
        print("left: " + str(left))
        print("right: " + str(right))
        print("top: " + str(top))
        print("bottom: " + str(bottom))
        self.win.destroy()

# Create Tkinter window
cali = tk.Tk()
app = Caliexperiment(cali, "Calibration")   # Create the calibration GUI

has_object = False
object_moving = False
object_moving_thread_started = False
object_detected_time = None
```

```
object_moving_end_time = None
remove_object = False
is_image_displayed = False
image_display_timer = None
image = None
spoiled_image_displayed = False
display_timer = None
spoiled_image = None
countdown_running = False
robotic = False
wait_message_displayed = False
spoiled = True
Normal = True
spoil = True
csv_path = 'C:/Users/UrbanLab/PycharmProjects/pythonProject/venv/uArm-
Python-SDK-2.0 (1)/uArm-Python-SDK-2.0/IMS mapping/excel_file/csv_file'
transfer_folder =
'C:/Users/UrbanLab/PycharmProjects/pythonProject/venv/uArm-Python-SDK-2.0
(1)/uArm-Python-SDK-2.0/IMS mapping/excel_file'  # Store the path
file_name = 'Test round_'
file_path = os.path.join(transfer_folder, file_name)
object_coordinate = []
sorted_coordinate = []
result_matrix = []
position_list = []
label_list = []
text_position = []
text_fresh_position = []
times = 0
check = True
TMA = True
check_carryover = True
actual_distance = 0
drifttime = np.zeros(3200)
intensity = np.zeros(3200)
recording = False


# Create the analysis GUI
root = tk.Tk()
root.title(" ")
root.state("zoomed")

#   Show the logo on the GUI
def display_image(image_path):
    original_image = Image.open(image_path)
    processed_image = original_image.convert("RGBA")
    # Get image transparency information
    data = processed_image.getdata()
    new_data = []
    for item in data:
        # If the background is white, make it transparent
        if item[0] == 255 and item[1] == 255 and item[2] == 255:
```

```
            new_data.append((255, 255, 255, 0))
        else:
            new_data.append(item)
    # Set new transparency information
    processed_image.putdata(new_data)
    origin_width, origin_height = original_image.size
    aspect_ratio = origin_width / origin_height
    new_width = 100
    new_height = int(new_width/aspect_ratio)
    processed_image = processed_image.resize((new_width, new_height))

    # Convert the processed image to Tkinter’s PhotoImage class
    photo = ImageTk.PhotoImage(processed_image)
    PUlab = tk.Label(root, image=photo, width=new_width, height=new_height)
    PUlab.photo = photo  # Keep a reference to PhotoImage to prevent it 
from being garbage collected
    PUlab.place(x=30, y=20)

# Create a Canvas widget for displaying images
canvas_left = tk.Canvas(root, width=640, height=480)
canvas_left.place(x=30, y=150)
image_path = "C:/Users/UrbanLab/PycharmProjects/pythonProject/venv/uArm-
Python-SDK-2.0 (1)/uArm-Python-SDK-2.0/IMS mapping/icon.png"
display_image(image_path)
text = tk.Label(root, text="Computer Vision-Assisted Robotized Sampling")
text.config( font='Arial 40')
text.place(x=130, y=10)
text_label = tk.Label(root,  text="Real-time monitoring video")
text_label.config(font='Arial 20')
text_label.place(x=180, y=90)
starttime = None

info_text = tk.Text(root, height=3, width=35)
info_text.config(font='Arial 16')
info_text.place(x=720, y=630)
status_text = tk.Text(root, height=2, width=30)
status_text.config(font='Arial 16')
status_text.place(x=100, y=640)
mode_text = tk.Text(root, height=2, width=13)
mode_text.config(font='Arial 16')
mode_text.place(x=500, y=640)

lock = threading.Lock()  # In threading, lock is used to control 
threading’s access to common information。

# Send  order function to Arduino
def send_command(command):
    ser.write(command.encode())
    ser.flush()  # Clear serial port buffer

# Measure the distance from the object
def lasersensor():
    global actual_distance
```

```
    send_command(str(6))
    data_list=[]
    data=[]
    starttime=time.time()
    while time.time()-starttime<5:
        line = ser.readline().decode('UTF-8').strip()
        data_list.append(line)
        for n in data_list:
            if n.strip():
                integer=int(n)
                data.append(integer)
    average=round(sum(data)/len(data))
    actual_distance = int(0.781*average+39.496)
    message = "Distance from the object: "+str(actual_distance) +" mm"
    display_info(message)

def display_info(message):
    info_text.insert(tk.END, message + "\n")
    info_text.see(tk.END)   # Automatically scroll to latest news

def info_display(status):
    status_text.insert(tk.END, status + "\n")
    status_text.see(tk.END)

def mode_display(mode):
    mode_text.insert(tk.END, mode + "\n")
    mode_text.see(tk.END)

def set_object_moving(value):
    global object_moving, object_moving_thread_started
    with lock:  # Automatically acquire and release lock
        object_moving = value
    time.sleep(0.5)
    object_moving_thread_started = False

#   Set the countdown alarm
def countdown(count):
    global object_moving, countdown_running
    if object_moving or not countdown_running:
        countdown_running = False
        return
    if count >= 0:
        status = 'Start sampling in: ' + str(count) + ' s'
        info_display(status)
        root.after(1000, countdown, count - 1)
    else:
        countdown_running = False

def start_countdown():
    global countdown_running
    if not countdown_running:
        countdown_running = True
        countdown(5)
```

```
def set_image_display_timer():
    global image_display_timer
    if image_display_timer:
        root.after_cancel(image_display_timer)
    image_display_timer = root.after(5000, close_image)

def close_image():
    global is_image_displayed, image
    if is_image_displayed:
        canvas_left.delete("image")
        is_image_displayed = False

def display_spoilage_timer():
    global display_timer
    if display_timer:
        root.after_cancel(display_timer)
    display_timer = root.after(5000, close_spoiled_image)

def close_spoiled_image():
    global spoiled_image_displayed, spoiled_image
    if spoiled_image_displayed:
        canvas_left.delete("spoiled_image")
        spoiled_image_displayed = False

# Check is there any carryover in the instrumental blank
def carry_or_not():
    global check
    max_intensity = 0
    x_axis = np.zeros(3200)
    y_axis = np.zeros(3200)
    arduino.write(b'1')
    k = 0
    while k < 3200:
        b = arduino.readline()
        b = b.decode()
        b = b.strip()
        values = b.split(",")
        if len(values) == 2:
            x_axis[k] = float(values[0])
            y_axis[k] = float(values[1])
            k += 1

    match_indices = []
    for x_idx, x_number in enumerate(x_axis):
        if str(RIP_value*1000-100) <= str(int(x_number))[:3] <=
str(RIP_value*1000+100):
            match_indices.append(x_idx)
    while True:
        for idx, number in enumerate(y_axis):
            if idx not in match_indices:
                if number > 500:
                    message = "Has carryover"
```

```
                    display_info(message)
                    check = False
                    break
            if check:
                message = "No carryover"
                display_info(message)
            check = True
            break

    matplotlib.use("TkAgg")
    fig, ax = plt.subplots()
    backend = plt.get_backend()
    if backend == 'TkAgg':
        manager = plt.get_current_fig_manager()
        manager.set_window_title('Spectrum')
        manager.window.geometry("560x420+670+180")
    ax.plot(x_axis / 1000, y_axis * 5 / 1024, linestyle='-', color='blue',
marker='o', markersize=0, linewidth=2)
    title_font = fm.FontProperties(weight='bold')
    plt.xlabel('Drift time / ms', fontproperties=title_font)
    plt.ylabel('Voltage / mV ', fontproperties=title_font)
    plt.title('Full spectrum', fontproperties=title_font)
    plt.xlim(0, 20)
    plt.ylim(0, 5.5)
    plt.show(block=False)  # Optional: Show the figure in non-blocking mode
    plt.pause(3)  # Wait a bit to ensure the figure is drawn
    plt.close(fig)  # Close the current figure
    Carry_button.config(state=tk.NORMAL)

def clean_signal():
    Carry_button.config(state=tk.DISABLED)
    Carry_or_not = threading.Thread(target=carry_or_not)
    Carry_or_not.start()

def Normal_mode():
    global Normal
    Normal = True
    Normal_button.config(state=tk.DISABLED)
    Spoiled_button.config(state=tk.NORMAL)
    mode = "General mode"
    mode_display(mode)

def Spoiled_mode():
    global Normal
    Normal = False
    Normal_button.config(state=tk.NORMAL)
    Spoiled_button.config(state=tk.DISABLED)
    mode = "Spoilage mode"
    mode_display(mode)

def folder_choose():
    global transfer_folder
    root.dir = fd.askdirectory(initialdir=r'D:/IMS data file/Jing
```

```
Chi/2024')
    transfer_folder = root.dir

Normal_button = tk.Button(root, text="General", background='sky blue', 
fg='black',command=Normal_mode)
Normal_button.config(font=('Arial', 16, 'bold'))
Normal_button.place(x=800, y=90)
Spoiled_button = tk.Button(root, text="Spoilage", background='sky blue', 
fg='black',command=Spoiled_mode)
Spoiled_button.config(font=('Arial', 16, 'bold'))
Spoiled_button.place(x=900, y=90)
folder_button = tk.Button(root, text="Folder", background='sky blue', 
fg='black',command=folder_choose)
folder_button.config(font=('Arial', 16, 'bold'))
folder_button.place(x=1010, y=90)
Carry_button = tk.Button(root, text="Clean", background='sky blue', 
fg='black',command=clean_signal)
Carry_button.config(font=('Arial', 16, 'bold'))
Carry_button.place(x=720, y=90)

#   Perform the analysis progress
def experiment():
    global has_object, object_moving_thread_started, object_moving, 
object_detected_time, object_moving_end_time, label_list, Normal, \
        times, is_image_displayed, object_coordinate, remove_object, image, 
position_list, result_matrix, sorted_coordinate, file_path
    Normal_button.config(state=tk.DISABLED)
    Spoiled_button.config(state=tk.NORMAL)
    folder_button.config(state=tk.NORMAL)
    Carry_button.config(state=tk.NORMAL)
    mode = "General mode"
    mode_display(mode)
    cap = cv2.VideoCapture(0)
    ret, frame1 = cap.read()
    roi1 = frame1[top:bottom, left:right]
    roi1_gray = cv2.cvtColor(roi1, cv2.COLOR_BGR2GRAY)

    while not has_object:
        ret, frame2 = cap.read()
        frame2 = cv2.resize(frame2, (0, 0), fx=1, fy=1)
        frame3 = frame2.copy()
        roi2 = frame2[top:bottom, left:right]
        roi2_gray = cv2.cvtColor(roi2, cv2.COLOR_BGR2GRAY)
        roi2_gray = cv2.GaussianBlur(roi2_gray, (11, 11), 0)
        frame_delta = cv2.absdiff(roi1_gray, roi2_gray)
        _, thresh = cv2.threshold(frame_delta, 50, 255, cv2.THRESH_BINARY)
        kernel = np.ones((5, 5), np.uint8)
        thresh = cv2.morphologyEx(thresh, cv2.MORPH_CLOSE, kernel)
        contours, _ = cv2.findContours(thresh.copy(), cv2.RETR_EXTERNAL, 
cv2.CHAIN_APPROX_SIMPLE)

        # parse results
        result = model(roi2)
```

```
        predictions = result.pred[0]
        boxes = predictions[:, :4]  # x1, y1, x2, y2
        scores = predictions[:, 4]

        if contours:
            if not object_moving_thread_started:
                object = threading.Thread(target=set_object_moving, 
args=(True,))
                object.start()
                object_moving_thread_started = True    # Change to True 
first and after the object is executed change to False
                #  Then, use the if conditional statement to avoid 
repeatedly enabling set_object_moving
        else:
            if not object_moving_thread_started:
                object = threading.Thread(target=set_object_moving, 
args=(False,))
                object.start()
                object_moving_thread_started = True

        for box in boxes:
            x1, y1, x2, y2 = map(int, box)
            x1 += left
            x2 += left
            y1 += top
            y2 += top
            object_x = int((x1 + x2) / 2)
            object_y = int((y1 + y2) / 2)
            frame2[top:bottom, left:right] = roi2
            width = int(x2 - x1)
            height = int(y2 - y1)
            area = width * height
            if area > 500 and area <100000:
                cv2.rectangle(frame2, (x1, y1), (x2, y2), (0, 255, 0), 2)
                cv2.circle(frame2, (object_x, object_y), 4, (0, 255, 0), -
1)
                if any(abs(i - object_x) <= 5 or abs(i - object_y) <= 5 for 
i in object_coordinate):
                    if not remove_object:
                        status = 'Please remove the object'
                        info_display(status)
                        remove_object = True
                elif any(abs(i - object_x) >= 5 or abs(i - object_y) >= 5 
for i in object_coordinate):
                    if object_moving:
                        object_moving_end_time = None
                    # object is not moving and object_moving continues to 
be False for more than 5 seconds
                    # Then performing the following operations
                    elif not object_moving and object_moving_end_time is 
None:
                        object_moving_end_time = time.time()
                        start_countdown()  # Start countdown timer
```

```
                        status = 'Something is moving'
                        info_display(status)
                    elif not object_moving and time.time() - 
object_moving_end_time >= 5:
                        Normal_button.config(state=tk.DISABLED)
                        Spoiled_button.config(state=tk.DISABLED)
                        Carry_button.config(state=tk.DISABLED)
                        object_moving = True  # Stop the countdown
                        frame3 = frame3[top: bottom, left:right]
                        image_file_path = file_path + '_' + str(times) + 
'.png'
                        cv2.imwrite(image_file_path, frame3)    # Save the 
picture
                        message = 'Save: ' + file_name + str(times) + 
'.png'
                        display_info(message)
                        has_object = True
                        status = 'Start sampling'
                        info_display(status)
                        cap.release()
                        cv2.destroyAllWindows()
                        canvas_left.delete("photo")
                elif object_moving:
                    object_detected_time = None
                    status = 'Something is moving'
                    info_display(status)
                elif not object_moving and object_detected_time is None:
                    object_detected_time = time.time()
                    start_countdown()
                elif not object_moving and time.time() - 
object_detected_time >= 5:
                    Normal_button.config(state=tk.DISABLED)
                    Spoiled_button.config(state=tk.DISABLED)
                    Carry_button.config(state=tk.DISABLED)
                    object_moving = True
                    frame3 = frame3[top: bottom, left:right]
                    image_file_path = file_path + '_' + str(times) + '.png'
                    cv2.imwrite(image_file_path, frame3)    # Save the 
picture
                    message = 'Save: ' + file_name + str(times) + '.png'
                    display_info(message)
                    has_object = True
                    status = 'Start sampling'
                    info_display(status)
                    cap.release()
                    cv2.destroyAllWindows()


        # Convert OpenCV image to Tkinter PhotoImage object
        frame2_tk = 
ImageTk.PhotoImage(image=Image.fromarray(cv2.cvtColor(frame2, 
cv2.COLOR_BGR2RGB)))
        canvas_left.create_image(0, 0, anchor=tk.NW, image=frame2_tk,
```

```
tags="photo")

        roi1_gray = roi2_gray
        canvas_left.update()

        # Add Tkinter event handling
        root.update_idletasks()
        root.update()

    cap.release()
    cv2.destroyAllWindows()
    canvas_left.delete("photo")
    Normal_button.config(state=tk.DISABLED)
    Spoiled_button.config(state=tk.DISABLED)
    folder_button.config(state=tk.DISABLED)
    Carry_button.config(state=tk.DISABLED)

    # Read the save picture and record the position of objects
    image_file_path = file_path + '_' + str(times) + '.png'
    img = cv2.imread(image_file_path)
    mmm = cv2.imread(image_file_path)
    aaa = model(img)
    prediction = aaa.pred[0]
    box = prediction[:, :4]  # x1, y1, x2, y2
    score = prediction[:, 4]
    object_coordinate = []
    label_coordinate = []
    for i in box:
        if area > 500 and area < 100000:
            x1, y1, x2, y2 = map(int, i)
            cv2.rectangle(img, (x1, y1), (x2, y2), (0, 255, 0), 2)
            cv2.rectangle(mmm, (x1, y1), (x2, y2), (0, 255, 0), 2)
            label_coordinate.append(x1)
            label_coordinate.append(y1)
            object_x = int((x1 + x2) / 2)
            object_y = int((y1 + y2) / 2)
            cv2.circle(img, (object_x, object_y), 4, (0, 255, 0), -1)
            cv2.putText(img, "Target", (x1, y1 - 10),
cv2.FONT_HERSHEY_SIMPLEX, 0.5, (0, 0, 0), 2)
            message = "object coordinate(x, y):" + "(" + str(object_x) +
"," + str(object_y) + ")"
            display_info(message)
            object_coordinate.append(object_x)
            object_coordinate.append(object_y)
    image = ImageTk.PhotoImage(image=Image.fromarray(cv2.cvtColor(img,
cv2.COLOR_BGR2RGB)))
    canvas_left.create_image(0, 0, anchor=tk.NW, image=image, tag="image")
    if Normal:
        cv2.imwrite(image_file_path, img)
    else:
        cv2.imwrite(image_file_path, mmm)
    is_image_displayed = True
    set_image_display_timer()
```

```
    message = "Total object number : " + str(int(len(object_coordinate) / 
2))
    display_info(message)
    sorted_coordinate = []
    sorted_coordinate = 
sorted(zip(object_coordinate[0::2],object_coordinate[1::2]))
    result_matrix = []
    for pair in sorted_coordinate:
        for num in pair:
            result_matrix.append(num)
    position_list = []
    for i in range(0, int(len(result_matrix)), 2):
        position = [result_matrix[i], result_matrix[i + 1]]
        position_list.append(position)
    aaa = []
    aaa = sorted(zip(label_coordinate[0::2], label_coordinate[1::2]))
    bbb = []
    for pair in aaa:
        for num in pair:
            bbb.append(num)
    label_list = []
    for i in range(0, int(len(bbb)), 2):
        ccc = [bbb[i], bbb[i + 1]]
        label_list.append(ccc)
    root.update_idletasks()
    root.update()

def robotic_move():
    global position_list,label_list, 
robotic,image_display_timer,starttime,file_name,times,is_image_displayed, 
spoiled, Normal, check_carryover, actual_distance
    robotic = True
    time.sleep(4)
    for i in range(0, int(len(position_list))):
        send_command(str(4))
        swift.set_position(x=(((int(position_list[i][1])) / (bottom - top)) 
* 185 + 100),
                           y=(((int(position_list[i][0])) / (right - left)) 
* 346 - 171.5), z=84)
        time.sleep(2)
        status = 'Measure the distance from object'
        info_display(status)
        laser = threading.Thread(target=lasersensor)
        laser.start()
        time.sleep(10)
        send_command(str(2))
        send_command(str(3))
        aaa = int(125-actual_distance)
        swift.set_position(z=aaa)
        status = 'Sampling'
        info_display(status)
        time.sleep(15)
        IMS_record = threading.Thread(target=IMS_signal)
```

```
        IMS_record.start()
        time.sleep(5)
        swift.set_position(z=100)
        send_command(str(5))
        status = 'Flushing with the nitrogen gas'
        info_display(status)
        swift.set_position(x=100, y=180, z=100)
        send_command(str(1))
        starttime = time.time()
        matplotlib.use("TkAgg")
        fig, ax = plt.subplots()
        backend = plt.get_backend()
        if backend == 'TkAgg':
            manager = plt.get_current_fig_manager()
            manager.set_window_title(file_name + str(times) + 'compound' +
str(i + 1))
            manager.window.geometry("560x420+670+180")
        data_process(ax)
        if Normal:
            plt.pause(10)  # Wait a bit to ensure the figure is drawn
            plt.close(fig)  # Close the current figure
            sleep = 15 - int(time.time() - starttime)
            if sleep > 0:
                time.sleep(15 - int(time.time() - starttime))
        else:
            spoiled = False
            plt.pause(10)  # Wait a bit to ensure the figure is drawn
            plt.close(fig)  # Close the current figure
            sleep = 15-int(time.time() - starttime)
            if sleep>0:
                time.sleep(15 - int(time.time() - starttime))


    swift.set_position(x=100, y=180, z=100)
    position_list = []
    label_list = []
    time.sleep(2)
    robotic = False

def wait_for_csv():
    global wait_message_displayed
    csv_files = []
    while True:
        for file in os.listdir(csv_path):
            if file.endswith('.mea'):
                csv_files.append(file)
            if csv_files and recording:
                break
            if not wait_message_displayed:
                message = "Data processing..."
                display_info(message)
                wait_message_displayed = True
            time.sleep(1)
```

```
        break

def IMS_signal():
    global drifttime, intensity, recording
    drifttime = np.zeros(3200)
    intensity = np.zeros(3200)
    arduino.write(b'2')
    k = 0
    while k < 3200:
        b = arduino.readline()
        b = b.decode()
        b = b.strip()
        values = b.split(",")
        if len(values) == 2:
            drifttime[k] = float(values[0])
            intensity[k] = float(values[1])
            k += 1
    recording = True

def data_process(ax):
    global wait_message_displayed, starttime, Normal, transfer_folder,
RIP_value, TMA, drifttime, intensity, recording
    wait_for_csv()
    recording = False

    if Normal:
        pass
    else:
        RIP = []
        for ms_idx, ms_number in enumerate(drifttime):
            if str(float(RIP_value) - 0.34) == str((ms_number))[:4]:
                RIP.append(ms_idx)
        while True:
            for idx, number in enumerate(intensity):
                if idx in RIP:
                    if number > 300:
                        TMA = False
                        break
            break

    # Draw scatterplots using Matplotlib
    ax.plot(drifttime / 1000, intensity * 5 / 1024, linestyle='-',
color='blue', marker='o', markersize=0, linewidth=2)
    title_font = fm.FontProperties(weight='bold')
    plt.xlabel('Drift time / ms', fontproperties=title_font)
    plt.ylabel('Voltage / mV', fontproperties=title_font)
    plt.title('Full spectrum', fontproperties=title_font)
    plt.xlim(0, 20)
    plt.ylim(0, 5)
    plt.show(block=False)

    # Transfer the file to selected file
    desktop_path = os.path.expanduser(transfer_folder)
```

```
    mea_file = []
    for f in os.listdir(csv_path):
        if f.endswith(".mea"):
            mea_file.append(f)

    try:
        for file in mea_file:
            mea_path = os.path.join(csv_path, file)
            destination_path = os.path.join(desktop_path, file)
            shutil.move(mea_path, destination_path)
    except FileNotFoundError:
        message = f"can not find the file"
        display_info(message)
    except Exception as e:
        message = f"error when moving file: {str(e)}"
        display_info(message)

    wait_message_displayed = False


def watch():
    global robotic,
position_list,spoiled,Normal,is_image_displayed,spoiled,file_path, times,
spoiled_image, TMA, text_position, text_fresh_position, label_list,
spoiled_image_displayed
    if Normal:  # Show the stream video
        cap = cv2.VideoCapture(0)
        while robotic:
            ret, frame = cap.read()
            watch =
ImageTk.PhotoImage(image=Image.fromarray(cv2.cvtColor(frame,
cv2.COLOR_BGR2RGB)))
            canvas_left.create_image(0, 0, anchor=tk.NW, image=watch,
tags="watch")
            canvas_left.update()
            root.update_idletasks()
            root.update()
        cap.release()
        cv2.destroyAllWindows()
        canvas_left.delete("watch")
    else:   # Show the stream video, then label the meat freshness on the
picture and show in the GUI
        text_position = []
        text_fresh_position = []
        for i in range(0, int(len(label_list))):
            cap = cv2.VideoCapture(0)
            while spoiled:
                ret, frame = cap.read()
                watch =
ImageTk.PhotoImage(image=Image.fromarray(cv2.cvtColor(frame,
cv2.COLOR_BGR2RGB)))
                canvas_left.create_image(0, 0, anchor=tk.NW, image=watch,
tags="watch")
```

```
                canvas_left.update()
                root.update_idletasks()
                root.update()
            cap.release()
            cv2.destroyAllWindows()
            canvas_left.delete("watch")
            image_file_path = file_path + '_' + str(times) + '.png'
            img = cv2.imread(image_file_path)
            for j in range(0, int(len(label_list))):
                if not TMA and j == i:
                    cv2.putText(img, "Spoiled", (label_list[i][0],
label_list[i][1] - 10), cv2.FONT_HERSHEY_SIMPLEX, 0.5, (0, 0, 0), 2)
                    if text_position:
                        for h in range(0, len(text_position), 2):
                            cv2.putText(img, "Spoiled", (text_position[h],
text_position[h + 1]), cv2.FONT_HERSHEY_SIMPLEX, 0.5, (0, 0, 0), 2)
                    if text_fresh_position:
                        for h in range(0, len(text_fresh_position), 2):
                            cv2.putText(img, "Fresh",
(text_fresh_position[h], text_fresh_position[h + 1]),
cv2.FONT_HERSHEY_SIMPLEX, 0.5, (0, 0, 0), 2)
                    text_position.append(label_list[i][0])
                    text_position.append(label_list[i][1] - 10)
                elif j == i:
                    cv2.putText(img, "Fresh", (label_list[i][0],
label_list[i][1] - 10), cv2.FONT_HERSHEY_SIMPLEX, 0.5, (0, 0, 0), 2)
                    if text_position:
                        for h in range(0, len(text_position), 2):
                            cv2.putText(img, "Spoiled", (text_position[h],
text_position[h + 1]), cv2.FONT_HERSHEY_SIMPLEX, 0.5, (0, 0, 0), 2)
                    if text_fresh_position:
                        for h in range(0, len(text_fresh_position), 2):
                            cv2.putText(img, "Fresh",
(text_fresh_position[h], text_fresh_position[h + 1]),
cv2.FONT_HERSHEY_SIMPLEX, 0.5, (0, 0, 0), 2)
                    text_fresh_position.append(label_list[i][0])
                    text_fresh_position.append(label_list[i][1] - 10)
                if j == int(len(label_list))-1:
                    ddd = file_path + '_' + str(times) + ' food freshness'
+ '.png'
                    cv2.imwrite(ddd, img)
            spoiled_image =
ImageTk.PhotoImage(image=Image.fromarray(cv2.cvtColor(img,
cv2.COLOR_BGR2RGB)))
            canvas_left.create_image(0, 0, anchor=tk.NW,
image=spoiled_image, tag="spoiled_image")
            spoiled_image_displayed = True
            display_spoilage_timer()
            TMA = True
            spoiled = True
            root.update_idletasks()
            root.update()
```

```
def quit_fullscreen():
    global has_object
    has_object = True
    root.attributes('-fullscreen', False)
    root.destroy()

exit_button = tk.Button(root, text="Finish", command=quit_fullscreen)
exit_button.config(width=8, heigh=2, font='Arial 15')
exit_button.place(x=1170, y=640)

#   Repeat the analysis progress
def start_experiment():
    global has_object, object_moving_thread_started, object_moving, 
remove_object\
        , object_detected_time, object_moving_end_time, times, image, 
image_display_timer, is_image_displayed
    send_command(str(1))
    experiment()
    time.sleep(2)
    has_object = False
    object_detected_time = None
    object_moving_end_time = None
    object_moving = False
    object_moving_thread_started = False
    remove_object = False
    Robotic_move = threading.Thread(target=robotic_move)
    Robotic_move.start()
    watch()
    time.sleep(6)
    times += 1
    root.after(10, start_experiment)

root.after(0, start_experiment)
root.mainloop()
```
